# Copper recovery from printed circuit boards using an oxidant-free deep eutectic solvent: experimental and molecular dynamics approach

**DOI:** 10.1038/s41598-026-42446-7

**Published:** 2026-04-29

**Authors:** Pardis Bedrodian, Saeid Karimi, Mojtaba Esmailzadeh

**Affiliations:** 1 Department of Metallurgy and Materials Engineering, Hamedan University of Technology, Hamedan, Iran; 2 Department of Mechanical Engineering, Persian Gulf University, Bushehr, Iran

**Keywords:** Printed circuit board (PCB), Solvometallurgy, Choline chloride, Malonic acid, Molecular dynamic simulation, Chemistry, Environmental sciences, Materials science

## Abstract

PCB recycling is crucial due to the growing electronic waste, which poses significant environmental hazards from hazardous materials like heavy metals. Traditional recycling methods, involving strong acids and high temperatures, generate toxic chemical waste and release hazardous fumes. As a result, it is essential to investigate green alternatives to reduce these environmental hazards. This study investigates the recovery of copper from waste printed circuit boards (PCBs) using a green, oxidant-free two-component deep eutectic solvent (DES) composed of choline chloride and malonic acid (ChCl: MOA), without an oxidant agent. The influential parameters-temperature, leaching time, PCB/DES mass ratio, and agitation speed-were optimized using Taguchi method, revealing that higher temperatures and longer leaching durations significantly enhance copper extraction. Analysis of variance (ANOVA) confirmed that temperature, leaching time, and PCB/DES ratio as statistically significant factors, whereas agitation speed had a negligible impact within the studied range. According to the optimization evaluation, maximum copper recovery of 89.5% was achieved under conditions of 100 °C temperature, 360-min leaching time, 0.04 g/g PCB/DES ratio, and 200 rpm agitation speed. Complete recovery was observed for Ag, while Sn showed a moderate recovery of 77.2%, which attributed to its alloying with copper and its limited dissolution in DES. Cu^2+^ forms stable [CuCl_4_]^2–^ complexes in the ChCl: MOA DES via strong first-shell coordination with Cl^–^, as confirmed by molecular dynamics (MD) simulations and UV–Vis spectroscopy. RDF and coordination number analyses reveal a negligible interaction between Cu^2+^ and MOA. Additionally, hydrogen bonding is dominated by ChCl–Cl^–^ interactions, while MOA is primarily involved in intramolecular hydrogen bonding, limiting its contribution to solvation and metal coordination after leaching.

**Supplementary Information:**

The online version contains supplementary material available at 10.1038/s41598-026-42446-7.

## Introduction

Waste printed circuit boards (WPCBs) are an important part of electronic waste, which contain both valuable and harmful metals, so recycling them efficiently is important^1^. These boards comprise copper (up to 75% of the metallic content), iron (5.76%), zinc (5.76%), tin (4.87–11.31%), lead (7.74–14.86%), and trace amounts of gold and silver^2,3^. Firstly, electronic components were carefully removed before processing in order to maximize the recovery of the metal-rich substrate. The high economic value of metals like copper and gold makes WPCB recycling an industrial priority, while improper disposal of toxic metals, such as lead, risks contaminating soil and groundwater, posing environmental and health challenges^4^. Efficient recycling reduces reliance on mineral extraction, a costly and energy-intensive process, thereby highlighting the critical role of recycling in an era where energy conservation is a pressing challenge. By recovering metals such as copper and gold from WPCBs, recycling not only mitigates economic losses but also alleviates the environmental burden associated with mining activities^5^. According to the United States Environmental Protection Agency, approximately 85% of PCBs are disposed of through incineration or landfilling, highlighting the critical need to study and develop effective recycling methods for these materials^6,7^.

Over time, methods for recycling waste PCBs have significantly evolved. These improvements encompass various stages, such as pretreatment, employing physical, thermal, and chemical techniques, as well as metal recovery and purification through hydrometallurgical, pyrometallurgical, and bio-hydrometallurgical processes. Due to the complex composition of WPCBs, recycling them presents considerable challenges, making it essential to integrate the appropriate combination of technologies to achieve efficient and effective processing of this waste stream^8–10^.

Pyrometallurgical treatment of waste WPCBs involves high-temperature processing to recover metals, resulting in the formation of a copper-rich alloy phase and a slag phase containing oxides such as SiO_2_ and Al_2_O_3_. The alloy phase, which includes valuable metals like lead, tin, and precious metals, undergoes further refining through copper electrorefining to achieve pure copper^11^. However, this method presents significant environmental challenges, including the emission of toxic gases, high energy consumption, and severe pollution due to removing organic materials at elevated temperatures^12^. Additionally, a notable limitation of pyrometallurgy is the loss of substantial quantities of valuable metals such as zinc, lead, tin, and cadmium as fly ash, attributed to their high vapor pressures during processing^1,13^. These drawbacks highlight the need for improved or complementary recycling technologies to enhance metal recovery efficiency while minimizing environmental impact^11,14^. The hydrometallurgical process is a set of widely used techniques for recycling metals from waste PCBs. The main idea of this method is dissolving target metals in acids like H_2_SO_4_, HCl, HNO_3_, and CH_3_COOH, and in the presence of oxidants, like (NH_4_)_2_S_2_O_8_, and H_2_O_2_. After dissolving and concentrating, the dissolved metals are recovered using methods like precipitation or electrolysis. This method faces challenges due to high chemical consumption, secondary pollution, and the production of toxic waste^15^.

Given the drawbacks of conventional recycling techniques, such as pyrometallurgy and hydrometallurgy, which often generate toxic emissions and hazardous wastewater, there is a pressing need for more sustainable alternatives. Solvometallurgy, particularly using deep eutectic solvents (DESs), has emerged as a promising approach due to its environmentally friendly nature, high selectivity in metal extraction, and simple preparation process. This innovative method offers a greener and more efficient pathway for recovering valuable metals while minimizing ecological impact^16–18^.

A wide range of DESs have been investigated, predominantly based on choline chloride (ChCl) as the hydrogen bond acceptor, paired with different hydrogen bond donors such as ethylene glycol (EG), organic acids (e.g., malonic acid (MOA), citric acid (CA), lactic acid (LA)), and inorganic salts like CaCl_2_·6H_2_O^12,15^. Several studies have achieved high metal recovery, especially for copper and gold, by incorporating oxidants such as hydrogen peroxide (H_2_O_2_), iodine (I_2_), or metal chlorides (e.g., CuCl_2_·2H_2_O, FeCl_3_) into the DES system. For example, the ChCl–EG DES combined with CuCl_2_·2H_2_O enabled complete gold recovery (100%) at 90 °C in 120 min^19^. Similarly, H_2_O_2_-enhanced systems such as ChCl–EG and ChCl: FOR (formic acid) demonstrated efficient copper and silver leaching^20,21^. While various DESs have shown promise for metal recovery from PCBs, including those based on choline chloride paired with hydrogen bond donors like ethylene glycol, organic acids, and inorganic salts, many of these systems rely on the use of oxidants such as hydrogen peroxide, iodine, or metal chlorides to enhance recovery^22^. However, the incorporation of oxidants introduces significant limitations, including increased environmental impact, higher costs, handling hazards, and complications in downstream separation and reuse. Unlike traditional approaches, which often require these harsh reagents, this study focuses on a non-oxidant DES system (ChCl: MOA) to reduce the environmental and operational challenges while still achieving efficient metal recovery.

In this paper, we aim to explore an environmentally friendly approach for recovering copper from waste PCBs using a green, oxidant-free DES composed of choline chloride and malonic acid (ChCl: MOA). ChCl: MOA has also been shown to be highly effective for recovering valuable metals from secondary resources, such as spent lithium-ion batteries and industrial residues, with recovery efficiencies often exceeding 95%. For example, in lithium-ion battery cathodes, a DES combining ChCl and MOA achieved 98.61% cobalt and 98.78% lithium recovery at 100 °C^23^. In a comparative study, the ChCl: MOA DES outperformed malonic acid aqueous solutions alone for LiCoO_2_ recovery^24^. In the context of PCBs, studies have shown varying results, with Cu(II) extraction reaching up to 15.8 wt% after thermal pre-treatment of the PCB materials, as demonstrated by Łukomska et al.^25^. While effective for some elements, the extraction efficiency was lower compared to other methods, especially in the absence of additives like iodine. For PCBs, ChCl: MOA has shown moderate extraction efficiency, particularly for Cu and Sn, with recovery rates exceeding 75% under optimized conditions^26^. This study focuses on optimizing key leaching parameters to maximize copper extraction efficiency while minimizing environmental impact. Additionally, the leaching mechanisms have been investigated through structural, spectroscopic, and molecular dynamics analyses to provide a comprehensive understanding of metal dissolution and complexation in the DES system.

## Materials and methods

### Materials and characterization methods

All the PCBs used in this study were a mixture of components from various electronic devices (which are predominantly from old TVs), and no chemical pre-treatment was performed before the manual separation of the components. To facilitate the removal of components, the backside of the electronic boards was heated with a lighter. This caused the solder to melt, making it easier to detach the components such as resistors, integrated circuits (ICs), and chips. The PCBs were then shredded to particles < 5 mm using a shredding machine. To ensure representativity and address particle size concerns, the shredded material was screened through a 14-mesh sieve (particle size < 1680 μm). These fine particles were subsequently used for leaching tests and preliminary material analysis. For the analysis, 1 g of the screened material was dissolved in 30 mL of aqua regia (a 3:1 mixture of hydrochloric acid and nitric acid). The solution was heated to 100 °C and allowed to boil for 30 min, then filtered. The remaining residue was rinsed several times with deionized water, and the volume of the solution was adjusted to 100 mL. The final solution was then analyzed using Inductively Coupled Plasma (ICP) spectroscopy, as presented in Table 1. The sample contained copper as the major element at 8.82%, with notable amounts of Ca (1.69%), Sn (1.00%), and Ag (0.07%). This composition reflects the typical use of copper for electrical conductivity in PCBs, along with tin and silver from solder materials, while the presence of calcium may result from fillers or additives used in the board’s construction. ChCl (98.0% purity, ChCl) and MA (99.0% purity, C_3_H_4_O_4_) were acquired from Merck Co. and utilized without additional purification. Philips PW-3710 XRD equipment with CuK_α_ beam was used to identify the PCB phase compounds. The analysis was conducted at 0.02 degrees per second, covering an angle range from 10 to 90 °. The metal concentrations in the leach solutions was measured using an atomic absorption spectrometer (AAS, Varian 240). The morphology and chemical composition were analyzed using a scanning electron microscope (SEM) with an energy-dispersive X-ray spectrometer (EDS) (MIRa3 FEG-SEM TESCAN). The chemical structure of the synthesized DES before and after the leaching process was analyzed using a Fourier transform infrared spectrometer (FTIR), (PerkinElmer Spectrum version 10.02.00). The DESs before and after leaching of PCBs were analyzed using UV-Visible spectroscopy on a Shimadzu UV-Vis-1800 spectrophotometer.

**Table 1:** Chemical composition of PCBs.

Element	Ag	Ba	Ca	Cu	Fe	K	Mn
Concentration (mg/kg)	742	3530	16,900	88,157	5810	650	2920
Element	Na	Ni	Pb	S	Sn	Ti	Zn
Concentration (mg/kg)	1340	1470	1995	1320	10,010	1150	3450

### DES synthesis

DESs are classified into types based on the formula Cat^+^X^−^zY, where Cat^+^ is typically a choline cation in ChCl, X is a Lewis base, often a halide anion which is chloride in a ChCl molecule, Y is a Lewis or Brønsted acid, and z represents the number of Y molecules interacting with the anion. While MOA functions as a hydrogen bond donor (HBD), corresponding to Y^27^. The mol ratio of ChCl: MOA for the DES was chosen as 1:1, based on previous studies in the literature^25,28^. The synthesis was conducted in a glass vial placed in an oil bath at 70 °C for 1 h. It is essential to mention that to minimize the testing errors caused by the significant moisture absorption of these materials, particularly ChCl, all components were thoroughly dried in an oven at 70 °C for 3 h before synthesis.

### Leaching experiments based on the Taguchi design

The Taguchi experimental design method was employed to maximize the copper recovery. Given the number of variables and the desired levels selected (including 4 variables, each at 3 levels), an L_9_ orthogonal array was chosen to conduct the experiments. Three parameters, including the temperature range of 40 to 100 °C, leaching time in the range of 40 to 720 min, and PCB-to-DES mass ratio (PCB/DES) in the range of 0.02 to 0.12 g/g, were investigated. Table 2 shows the selected variables and their levels. The selection of parameters and their levels was based on previous studies to ensure relatively optimal conditions^29–32^.

**Table 2:** Variables and their levels for Taguchi optimization.

Level	T (°C)	Leaching time (min)	PCB/DES (g/g)	Agitation speed (rpm)
1	40	40	0.02	200
2	70	180	0.04	400
3	100	720	0.12	600

In the PCB leaching experiment, the following formula was used to determine the Cu recovery:

1 $${R_{Cu}}=\frac{{{C_{Cu}} \times V}}{{{G_{Cu}} \times M}} \times 100$$

where *R*_*Cu*_ is the Cu recovery (%), C_Cu_ is the Cu concentration in the leaching solution, V is the volume of the leaching solution (L), G_Cu_ is the copper grade in PCB (%), and M is the PCB mass (g). To determine the impact of disturbance or uncontrollable variables in the process, each experiment was conducted with the specified variables under identical conditions. Certain experiments were duplicated to confirm consistency, resulting in an overall error of less than 5%. All computations were cross-verified using DX13 software. Finally, through an analysis of variance (ANOVA), the statistical impact of each variable on Cu recovery was assessed. The validity and accuracy of the Taguchi predictions were examined by calculating the confidence interval and conducting a confirmation test (performing experiments under Taguchi’s optimal conditions).

The optimal leaching conditions were determined through Taguchi optimization method, which identified the parameters that maximize copper recovery efficiency for subsequent experimental studies conducted across temperature ranges of 40–110 °C, leaching time of 40–1440 min, and PCB/DES ratio of 0.01–0.12 g/g. It is worth noting that the experimental results exhibited a maximum error of less than 5%. The Taguchi method was used as a screening tool to identify the optimal parameters for copper recovery during PCB leaching in DES. This approach was chosen for its efficiency in reducing experimental trials while providing valuable insights into the key factors, such as temperature, leaching time, and PCB/DES ratio. Although the method evaluates parameters in discrete levels and doesn’t allow precise inter-level comparison, it effectively narrows down significant factors, which were then studied in greater detail to refine the conditions for maximum recovery. To verify the accuracy and consistency of the results, certain tests were conducted twice. This repetition was performed to ensure that the findings were reliable and could be consistently reproduced under the same conditions, minimizing any potential experimental errors or inconsistencies.

### Molecular dynamics simulations

Molecular dynamics (MD) simulations were performed to explore the interactions between the DES and Cu cations through radial distribution function (RDF) analysis. These simulations provide valuable insights into the coordination and solvation behaviors of Cu^2+^ ions in the DES medium, helping to explain the observed leaching behavior. The results from the MD simulations support the experimental findings by revealing how the DES components interact with Cu ions, thus rationalizing the efficiency of copper recovery observed in the leaching tests. This integration of simulations and experimental data offers a deeper understanding of the underlying mechanisms of metal extraction. The Universal force field was applied to all simulations using Material Studio (Version 2023). Geometry optimization was performed using the Forcite module with the Smart algorithm to achieve minimized energy configurations, setting convergence thresholds at 0.001 kcal/mol for energy and 0.001 kcal/mol/Å for force, with a maximum of 5000 iterations. Energy computations utilized the Universal force field, and atomic charges were assigned via the QEq method. Electrostatic interactions were calculated at the atomic level using a cubic spline truncation approach with a cutoff distance of 9.5 Å, spline width of 1 Å, and buffer width of 0.5 Å. Similarly, van der Waals forces were assessed atomically with the same cubic spline truncation parameters (cutoff 9.5 Å, spline width 1 Å, buffer width 0.5 Å). Subsequently, an amorphous simulation box was generated using Amorphous Cell Tools to house 329 molecular pairs of ChCl and MOA, together with 4 copper ions, according to the leaching optimization parameters described in Sect. Results of leaching experiments. The amorphous cell was constructed at a target density of 1.05 g/cm³ using 1000 loading steps and an initial temperature of 298 K. The geometry optimization was performed using the Smart algorithm, applying convergence criteria of 2 × 10^− 5^ kcal/mol for energy, 0.001 kcal/mol/Å for force, a atm for pressure, and 1 × 10^–5^ Å for displacement, with a limit of 10,000 iterations. For the energy calculations, the Universal force field was utilized along with the current charge assignments. During the cell optimization, the same parameters as in the geometry optimization were maintained for both electrostatic and van der Waals interactions^31^.

In the subsequent simulation steps, both the NVT ensemble (constant particle number, volume, and temperature) and the NPT ensemble (constant particle number, pressure, and temperature) were employed during the equilibration and production phases to achieve conditions of 373.15 K and 1 atm. The equilibration phase was divided into two segments: initially, a pre-NVT period lasting 1,000 ps utilizing velocity scaling as the thermostat, followed by an NVT phase of 1,000 ps employing the Nosé-Hoover-Langevin (NHL) thermostat. The integration timestep was set to 1.0 fs, and the total simulation time was 1,000 ps, with initial atomic velocities assigned randomly. For the potential energy calculations, the Universal force field was applied. The subsequent NVT simulation maintained identical conditions to the pre-NVT stage, running for 1,000 ps and using the velocities obtained from the pre-NVT run as starting values^33^. Once the system reached equilibrium, the simulation switched to the NPT ensemble to regulate pressure at 1 atm. The parameters for this stage included the NPT ensemble with temperature control via the NHL thermostat and a decay constant of 1.0 ps. Pressure was maintained at 1 atm using the Andersen barostat with a cell relaxation time constant of 1.0 ps. The timestep remained 1.0 fs, and the total simulation time was 1,000 ps. The Universal force field continued to be used for energy calculations. For both NVT and NPT simulations, the same settings applied during geometry optimization were used to handle electrostatic and van der Waals forces^34^.

**Fig. 1:**
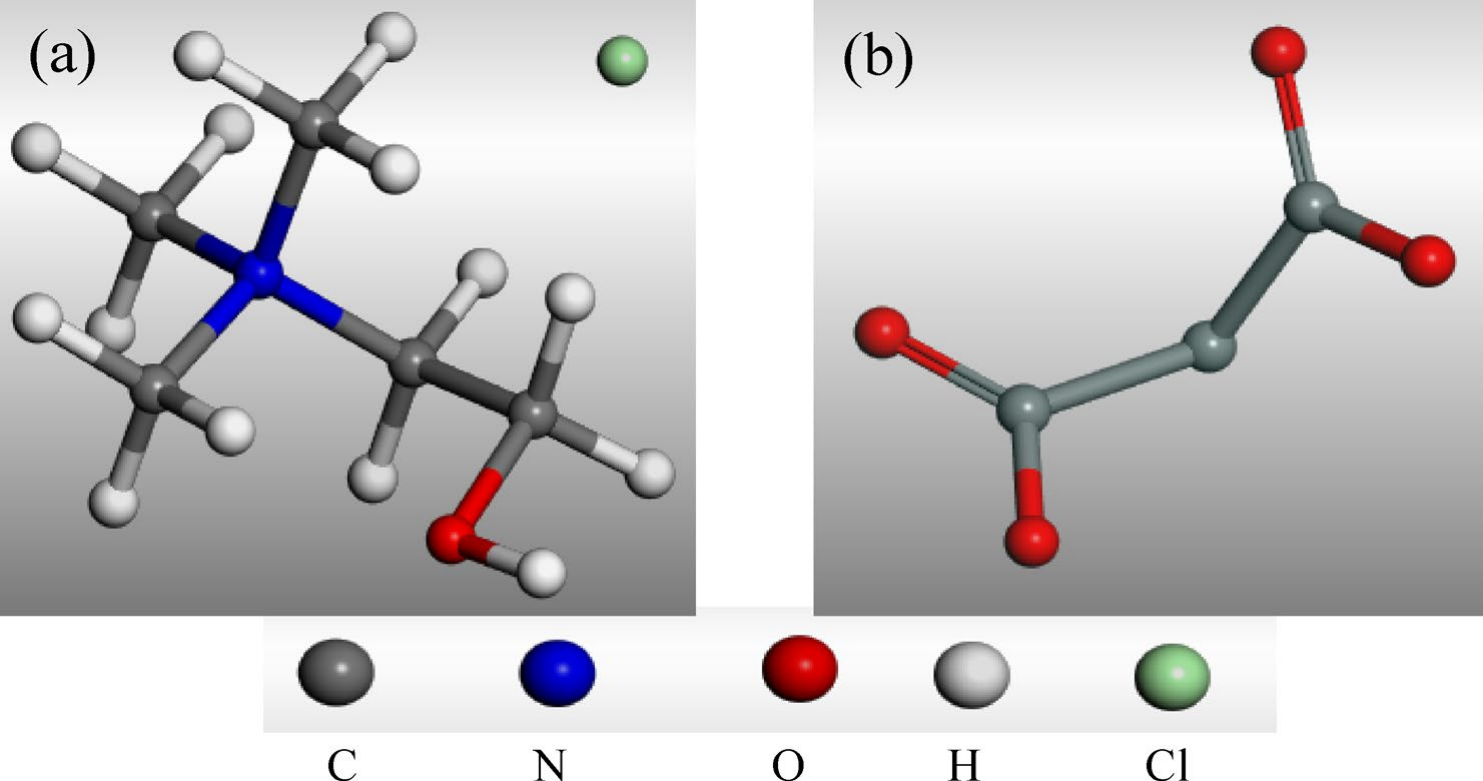
The molecular structure of **(a)** ChCl and **(b)** MOA.

In the concluding phase of MD simulation, RDF was employed to characterize the interactions between different anionic and cationic species by quantifying the interatomic center-to-center distances^30^. The RDF, denoted as *g*(*r*_*ij*_), represents the probability density of locating a particle of species “*i*” at a specific radial separation *r*_*ij*_ from a particle of species “*j*”. Consequently, this function effectively illustrates the extent of spatial correlation and interaction between a solute molecule and its adjacent solvent molecules, which indicates the formation and stability of cationic complexes within the system^35^. Figure 1 shows the ChCl and MOA molecules’ structures.

## Results

### Results of leaching experiments

**Fig. 2:**
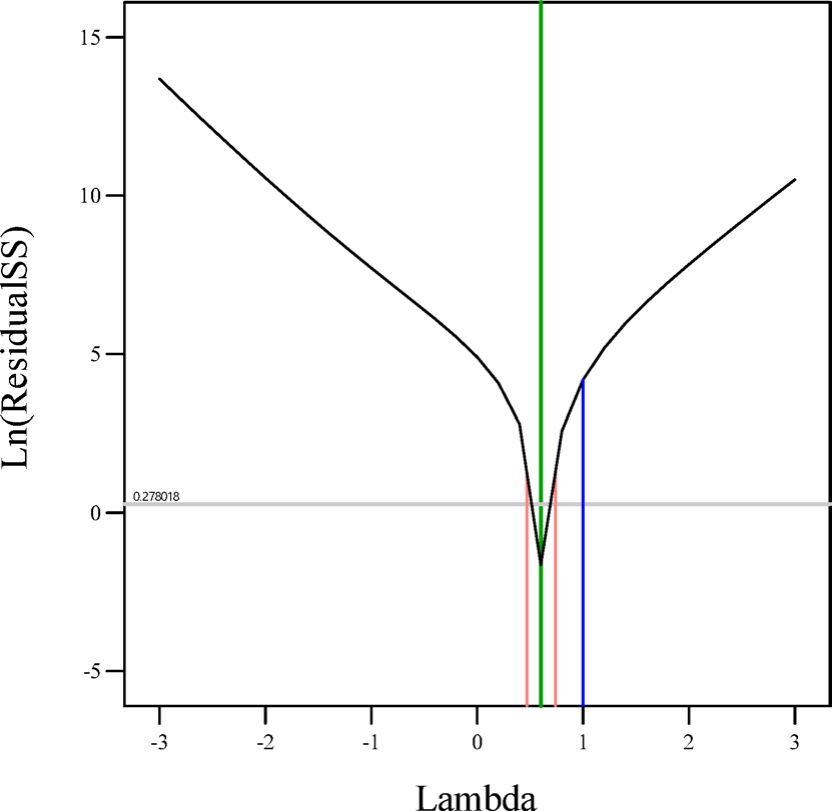
Box-Cox plot for copper recovery based on the Taguchi design.

Box-Cox graphs serve as a tool for determining the most suitable transformation function to use in the response variable. The lowest point in the Box-Cox plot indicates the optimal value of *λ*, resulting in the smallest residual sum of squares (SS) for the transformed model. Based on the Box-Cox graphs generated using the Taguchi method (Fig. 2), transforming λ^0.5^ for copper into the smallest residual sum of squares. This indicates the least error in the Taguchi method’s response.

The results of Cu recovery based on the L_9_ orthogonal array are presented in Table 3. Experiments were conducted twice for this purpose. Each run tests a unique combination of these parameters, and the resulting Cu recovery varies significantly, from as low as 2.9% to as high as 87.5%. The highest Cu recovery was achieved in Run 3, which used the highest temperature (100 °C) and the longest time (720 min), indicating that both high temperature and extended leaching time play a crucial role in maximizing copper recovery. The method effectively isolates variable impacts while reducing experimental runs compared to full factorial designs.

**Table 3:** Experiments based on the Taguchi design and Cu recovery.

Run	T (°C)	t (min)	PCB/DES (g/g)	Agitation speed (rpm)	Cu recovery (%)
1	100	40	0.12	400	21.5
2	70	40	0.04	600	14.2
3	100	720	0.04	200	87.5
4	100	180	0.02	600	75.6
5	40	180	0.04	400	14.6
6	40	40	0.02	200	2.9
7	70	720	0.02	400	48.9
8	40	720	0.12	600	7.4
9	70	180	0.12	200	14.3

Table 4 displays the ANOVA (Analysis of Variance) results for copper recovery during PCB leaching in a DES. At a 95% confidence level, the critical F-value for assessing statistical significance was determined as 19.3, based on 6 degrees of freedom for the tested variables and 2 for experimental error^36^. This benchmark F-value originates from standardized statistical tables. Variables with calculated F-values exceeding 19.3 are deemed statistically significant, indicating their meaningful impact on copper recovery efficiency in this experimental setup^37^.

The P-value method complements ANOVA by identifying statistically impactful variables. At a 95% confidence level, variables are deemed significant if their P-values fall below 0.05. The ANOVA analysis confirms the model’s robustness, with an exceptionally high F-value of 303.16 and a P-value of 0.0033, underscoring its reliability. Key factors influencing copper recovery include temperature (F-value: 556.81, P-value: 0.0018), leaching time (F-value: 223.49, P-value: 0.0045), and PCB/DES ratio (F-value: 129.20, P-value: 0.0077), all of which exceed the significance threshold. These findings emphasize that optimizing temperature, leaching time, and solvent ratio is critical for maximizing copper extraction efficiency, while agitation speed adjustments may not yield meaningful improvements under the studied conditions. The residual sum of squares (0.0628) with 2 degrees of freedom represents unexplained variability in the Cu recovery data, which includes the effect of agitation speed and experimental error. Since agitation speed was not statistically significant (*P* ≥ 0.05) within the tested range (200–600 rpm), its influence was incorporated into the residual term rather than being modeled separately^38^. This small residual value indicates that temperature, time, and PCB/DES ratio account for nearly all explainable variations (99.9% of the total variance), while agitation speed’s contribution was negligible under these experimental conditions. According to Taguchi optimization analysis, elevated temperature of 100 °C, extended leaching duration of 360 min, PCB/DES ratio of 0.02 g/g, and agitation speed of 200 rpm result in enhanced copper recovery. Following comprehensive parameter evaluation across a broad range, these conditions are established as the standard operating parameters.

**Table 4:** The results of the ANOVA for Cu recovery.

Source	Sum of Squares	df	Mean Square	F-value	*P*-value
Model	57.09	6	9.51	303.16	0.0033
A-Temperature	34.95	2	17.47	556.81	0.0018
B-time	14.03	2	7.01	223.49	0.0045
C-PCB/DES	8.11	2	4.05	129.20	0.0077
Residual	0.0628	2	0.0314		
Cor Total	57.15	8			

#### Effects of temperature

**Fig. 3:**
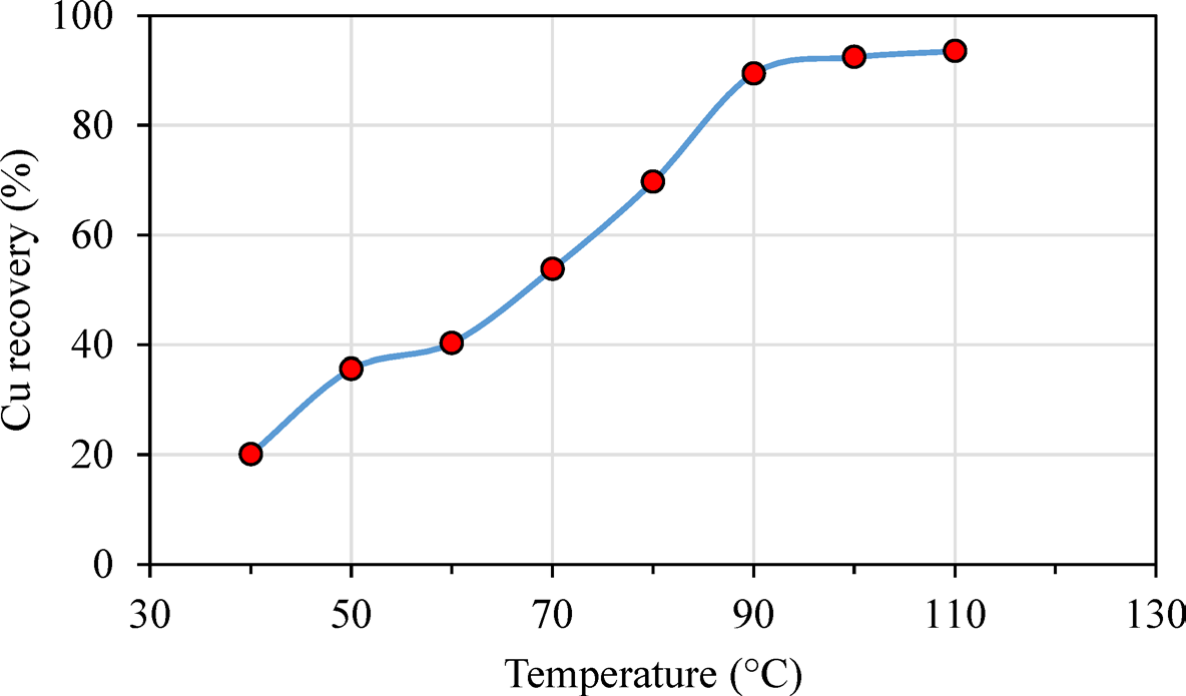
Effect of temperature on Cu recovery from PCB in the leaching time of 360 min, PCB/DES of 0.02 (g/g), and agitation speed of 200 rpm.

Figure 3 illustrates the effect of temperature on copper recovery from PCB leaching in the DES at a fixed leaching time of 360 min, a PCB/DES ratio of 0.02 g/g, and an agitation speed of 200 rpm. The results demonstrate a strong positive correlation between temperature and copper recovery efficiency across the studied temperature range of 40–110 °C. At lower temperatures, copper recovery is relatively modest, with approximately 20.1% recovery at 40 °C and 35.6% at 50 °C. As temperature increases to 60 °C, recovery reaches 40.3%, followed by a more substantial increase to 53.8% at 70 °C. The most significant improvements occur at higher temperatures, with recovery rates of 69.7% at 80 °C, 89.4% at 90 °C, and 92.4% at 100 °C. At the highest temperature tested (110 °C), copper recovery reaches 93.5%, representing only a marginal improvement over the 100 °C condition.

This temperature-dependent behavior demonstrates that higher temperatures significantly enhance the leaching efficiency through several mechanisms^39–42^. Elevated temperatures increase reaction rates according to the Arrhenius equation, improve mass transfer coefficients, and reduce solvent viscosity, all of which promote more effective dissolution of copper from the PCB matrix. The substantial increase in recovery between 70 °C and 100 °C suggests that this temperature range represents a critical threshold where the combined effects of enhanced kinetics and improved transport phenomena become particularly pronounced. The optimal temperature for copper recovery appears to be around 100 °C, where 92.4% recovery is achieved. While increasing temperature to 110 °C yields a slightly higher recovery (93.5%), th marginal improvement of only 1.1% points may not justify the additional energy costs and potential safety concerns associated with higher operating temperatures.

Several studies support the use of DESs for leaching metals from PCBs. Research by Łukomska et al.^25^ demonstrated that DESs such as ChCl and MOA can be used for extracting copper and other metals from waste PCBs, achieving low copper recovery (9.6 wt%) at a high temperature (1023 K) with a 7-hour thermal pre-treatment. In contrast, our study focuses on optimizing extraction at lower temperatures (around 100 °C) and examines a broader range of DES combinations, improving copper recovery efficiency and reducing environmental impact. Unlike their study, we also explore the influence of leaching time and PCB/DES ratio to optimize extraction conditions. Additionally, increasing temperature from 40 to 60 °C has been shown to improve copper recovery from PCB leaching in ChCl-oxalic acid DES^43^. As temperature increases, the kinetic energy of the system rises, reducing solvent viscosity and accelerating the dissolution of copper, which leads to greater leaching efficiency^12^.

#### Effects of leaching time

**Fig. 4:**
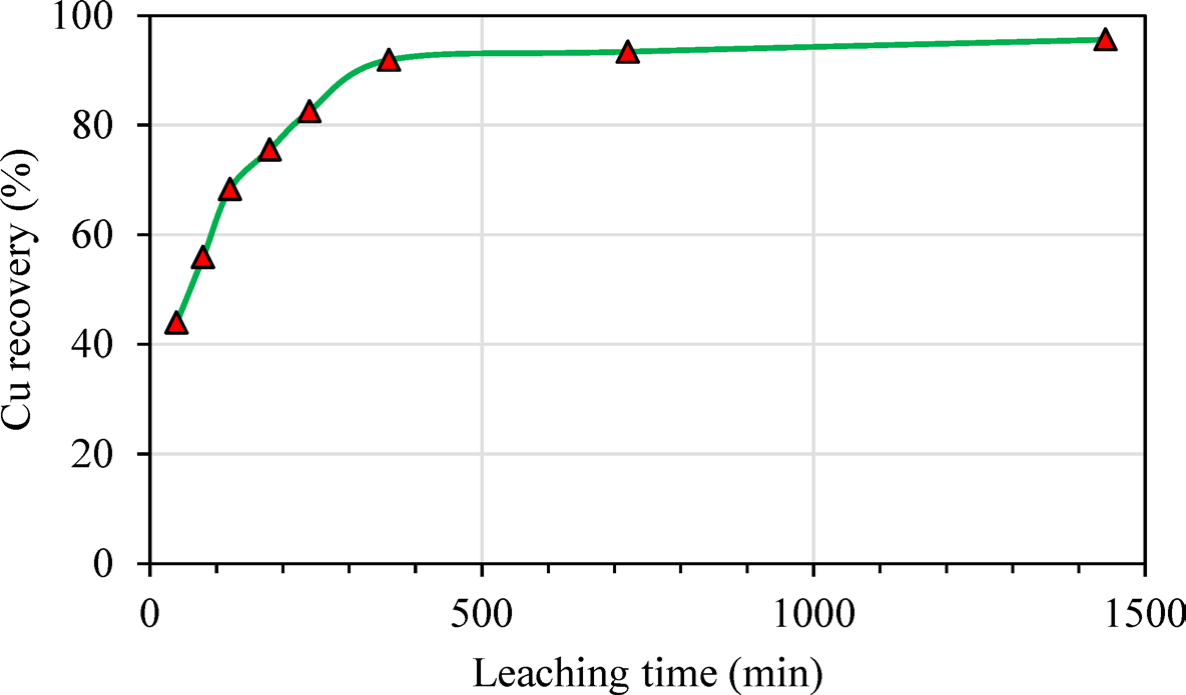
Effect of leaching time on Cu recovery from PCB in DES, temperature of 100 °C, and PCB/DES of 0.02 (g/g), and agitation speed of 200 rpm.

Figure 4 illustrates the impact of leaching time, varying from 40 to 1440 min, on copper recovery from PCB using DES. The leaching process was conducted at 100 °C with a PCB/DES ratio of 0.02 g/g and an agitation speed of 200 rpm. The results reveal a strong positive correlation between leaching time and copper recovery efficiency, with the recovery rate following a characteristic time-dependent pattern typical of solid-liquid extraction processes.

At shorter leaching times, copper recovery is moderate, with approximately 43.9% recovery at 40 min and 55.9% at 80 min. As leaching time extends to 120 min, recovery increases to 68.3%, followed by 75.5% at 180 min. The recovery continues to improve substantially with longer leaching times, reaching 82.5% at 240 min and 91.9% at 360 min. Extended leaching times of 720 min (12 h) yield 93.4% recovery, while the longest duration tested (1440 min–24 h) achieves 95.6% recovery. The time-dependent recovery behavior can be explained by the gradual dissolution kinetics of copper from the PCB matrix. Initially, easily accessible copper species are rapidly dissolved, resulting in relatively high recovery rates during the first few hours. As leaching progresses, the process becomes increasingly controlled by diffusion through the solid matrix and depletion of readily available copper sites, leading to a more gradual increase in recovery rates. The significant improvement from 240 min (82.5%) to 360 min (91.9%) indicates that 6 h represents a practical optimum for industrial applications, balancing efficiency with processing time. The optimal leaching time appears to be 360 min, where 91.9% copper recovery is achieved. While extending the leaching time to 720 min and 1440 min results in marginally higher recoveries (93.4% and 95.6%, respectively), the incremental improvements may not justify the significantly longer processing times and associated operational costs in industrial applications.

This trend aligns with research indicating that ChCl-based DESs are effective for metal recovery from electronic waste, with studies showing that choline chloride combined with malonic acid in a 1:1 ratio can successfully extract metals from solid waste phases^25^. The increasing recovery rate with time follows similar patterns observed in other research, where process factors such as leaching time significantly impact extraction efficiency^25^.

#### Effects of PCB/DES

**Fig. 5:**
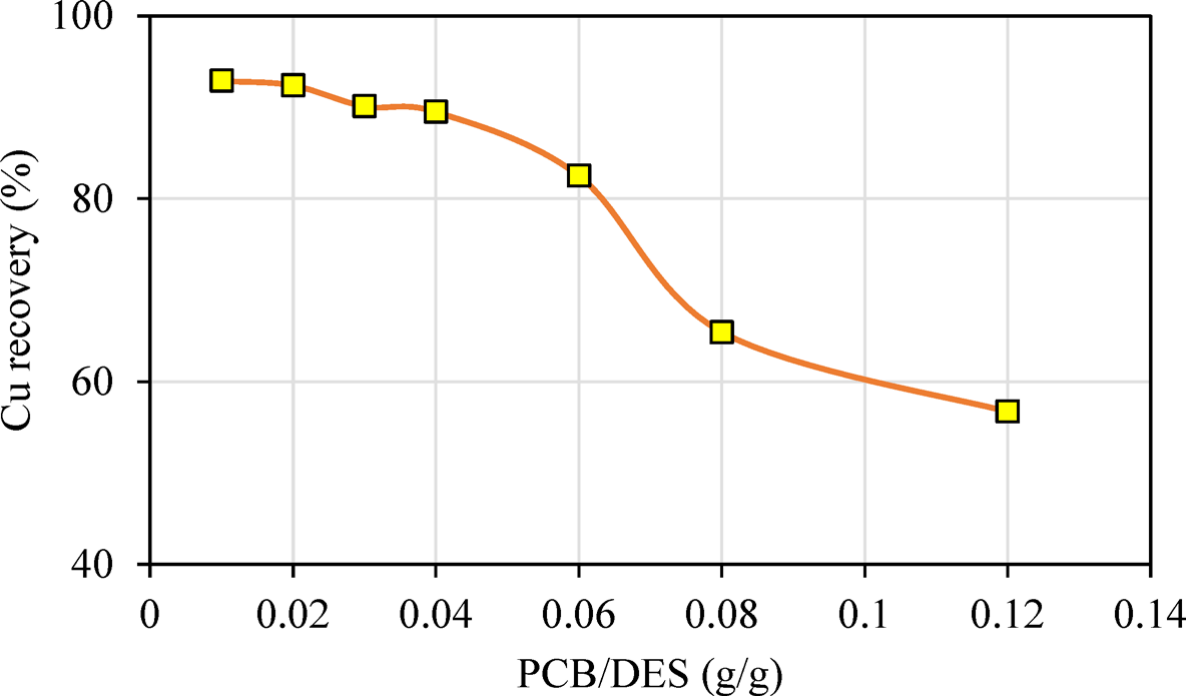
Effect of PCB/DES on Cu recovery from PCB in DES at 100 °C, and 360 min, and agitation speed of 200 rpm.

Figure 5 illustrates the impact of PCB/DES ratio on copper recovery from PCB leaching using DES at fixed conditions of 100 °C, 360 min leaching time, and 200 rpm agitation speed. The results demonstrate a clear inverse relationship between PCB/DES ratio and copper recovery efficiency across the studied range of 0.01 to 0.12 g/g. The minimal reduction in copper recovery observed between the 0.01 and 0.02 g/g ratios (from 92.9% to 92.4%) and the relatively small decrease to 0.04 g/g (89.5%) suggests that DES maintains high dissolution effectiveness within this range. However, the dramatic decline in recovery at higher ratios, particularly the 32.9% drop from 0.02 to 0.12 g/g, indicates that excessive PCB loading significantly impairs the leaching process.

This pattern suggests that lower PCB/DES ratios enhance the interaction between PCB particles and the solvent, facilitating better dissolution and more efficient extraction of copper. The decrease in Cu recovery with higher PCB/DES ratios during leaching is attributed to several factors: reduced solvent accessibility and increased system viscosity. At elevated ratios, the limited DES volume per unit PCB restricts effective contact between the solvent and metal surfaces, hindering dissolution^44^. Higher solid loading also raises viscosity, impairing reactant mobility and ion transfer. Additionally, excessive PCB content may saturate the solvent, reducing its leaching capacity, while particle agglomeration at dense ratios further limits diffusion^44,45^. These factors collectively diminish extraction efficiency as the PCB/DES ratio increases.

Based on the optimization analysis, the optimal conditions for copper recovery from PCB leaching in DES include a temperature of 100 °C, leaching time of 360 min, PCB/DES ratio of 0.04 g/g, and agitation speed of 200 rpm, yielding 89.5% copper recovery. These parameters represent the optimal balance between extraction efficiency and economic considerations. Table 5 summarizes the recovery of various elements from PCB leaching using ChCl: MOA DES under optimized conditions. Complete recovery was observed for Ag, Fe, Mn, Na, Ni, S, and Zn, while Sn and Pb showed a moderate recovery of 77.2% and 66.6%, respectively. In contrast, aluminum (Al, 28.8%) and titanium (Ti, 6.1%) exhibited low recoveries, likely due to their presence as stable oxides that are not readily dissolved by ChCl: MOA DES, and tin’s low recovery is attributed to its alloying with copper (Cu_6_Sn_5_), as further characterized by XRD analysis in the next section. Calcium (41.8%) and barium (2.5%) also showed limited extraction under optimum conditions.

**Table 5:** Leaching recovery for different metals at optimum conditions: temperature of 100 °C, leaching time of 360 min, PCB/DES ratio of 0.04 g/g, and agitation speed of 200 rpm.

Element	Ag	Al	Ba	Ca	Cu	Fe	Mn
Recovery (%)	100	28.8	2.5	41.8	89.5	100	100
Element	Na	Ni	Pb	S	Sn	Ti	Zn
Recovery (%)	100	100	66.6	100	77.2	6.1	100

Table 6 presents a comparative summary of copper recovery efficiencies from PCBs using various DES systems, highlighting both oxidant-free and oxidant-assisted approaches, along with their optimal leaching conditions and key findings. ChCl–EG with copper chloride as an oxidant stands out for its ability to recover gold efficiently and its high reusability, making it a promising green alternative; however, it is less effective for copper and nickel extraction and relies on relatively high temperatures and using metal salts, which may complicate downstream processing^19^. In contrast, ChCl–EG combined with H_2_O_2_ enables rapid and energy-efficient copper recovery at room temperature, supporting low-carbon recycling. Still, its selectivity for other valuable metals is limited, and it may not be suitable for comprehensive recovery from complex e-waste streams^20^. Calcium chloride–ethylene glycol (CaCl_2_·6H_2_O–EG) systems, especially when paired with ultrasound or copper chloride, can selectively extract copper and accelerate leaching rates dramatically, overcoming viscosity and passivation issues^18,46^. ChCl–MOA and mixtures of DESs and ionic liquids (ILs) have shown potential for high silver extraction. Still, copper recovery remains low, and separating metals after leaching remains a technical challenge, highlighting the need for further process optimization^47^. Carboxylic acid-based DESs, such as those with formic, oxalic, and lactic acids, demonstrate efficient copper and moderate silver leaching, with selectivity strongly influenced by the acid component; for example, oxalic acid-based systems favor silver over copper due to precipitation phenomena, while lactic and formic acid systems are more balanced, though still less effective for gold^21^. The use of iodine as an oxidant in ChCl–AA systems enables the nearly complete recovery of copper, nickel, and gold, offering a sustainable and cost-effective alternative to conventional methods, but may introduce additional chemical handling and separation steps^6^. Across all studies, a key strength of DESs is their environmental friendliness, tunability, and potential for selective metal recovery; however, weaknesses include limited selectivity for certain metals, the need for process intensification (e.g., ultrasound), and challenges in post-leach separation and solvent recycling. This study achieved 89.5% copper recovery from PCBs using a ChCl: MOA DES, outperforming DES systems reliant on oxidizing agents like CuCl_2_, H_2_O_2_, or I_2_. While other non-oxidant DES formulations (e.g., ChCl: LA, and ChCl: FOR) showed lower Cu recovery after long leaching time, the ChCl: MOA system also achieved 100% Ag recovery and 77.2% Sn recovery, contrasting with oxidant-dependent methods where Sn recovery is often limited (< 77%) due to alloying with copper. Although ultrasound-assisted DES leaching accelerated kinetics, it still required CuCl_2_, whereas this work achieved comparable efficiency (100 °C, 360 min, 0.04 g/g PCB/DES). Environmentally, the absence of corrosive oxidants (e.g., H_2_O_2_, CuCl_2_) reduces chemical waste and hazards.

**Table 6:** Comparison of copper recovery from waste PCBs using different DES systems, optimum leaching conditions, oxidant usage, and important findings from recent studies.

Type of DES	Recovery (%)	Optimum Leaching Conditions	Type of Oxidant	Important findings	References
ChCl–EG	Au: 100 Cu: 48 Ni: 12.5	1.5 M CuCl_2_·2H_2_O, 90 °C, 120 min, PCB/DES 0.001 g/mL	CuCl_2_·2H_2_O	DES-CuCl_2_·2H_2_O enables green Au recovery from e-waste via hydrogen bonds/ligand coordination, with > 97% reusability.	^19^
ChCl–EG	Cu: 97.8 Ni = N.R. Au = N.R.	20 °C, 25 min, PCB/DES 0.01 g/mL, 5 wt% H_2_O_2_	H_2_O_2_	ChCl-EG DES with H_2_O_2_ enables low-carbon Cu recovery via electrodeposition, cutting CO_2_ by 93.6 kg/ton PCBs, advancing sustainable e-waste recycling.	^20^
CaCl_2_·6H_2_O–EG	Cu = N.R. Ni = N.R. Au = 0	1 M CuCl_2_, 50 °C, 60 min	CuCl_2_, FeCl_3_	CaCl_2_·6H_2_O–EG DES with CuCl_2_ selectively leaches copper from PCBs, enabling efficient gold and nickel recovery by filtration.	^18^
CaCl_2_·6H_2_O–EG	Cu = N.R. Ni = N.R. Au = 0	0.1 M CuCl_2_, 50 °C, 300 W/cm^2^ power ultrasound	CuCl_2_	Ultrasound in CaCl_2_·6H_2_O–EG DES accelerates copper leaching from PCBs by 10,000 times, overcoming viscosity limitations and passivation.	^46^
ChCl–MOA	Cu = 15.7 Ni = N.R. Au = N.R.	60 °C	H_2_O_2_	Mixture of DESs and ILs achieve high metal extraction (e.g., 100% Ag) from pre-treated WPCB powder, but post-leach metal separation remains a challenge.	^47^
ChCl–EG	Cu = 4.2 Ni = N.R. Au = N.R.				
ChCl: AA	Cu = 99 Ni = 92 Au = 90	65 °C, 300 rpm, S/L ratio 10 g/L, 96 h, 0.1 mol/L I_2_	Iodine (I_2_)	The process offers a sustainable, environmentally friendly, and cost-effective alternative to conventional hydrometallurgical and pyrometallurgical methods for recovering critical and precious metals from e-waste	^6^
ChCl: EG	Cu = 96 Ni = 50 Au = 0				
ChCl: MOA	Cu = 89 Ni = 89 Au = 0				
ChCl: CA	Cu = 99 Ni = 94 Au = 93				
ChCl: LA	Cu = 86 Ni = 66 Au = 0				
ChCl: FOR	Cu = 75 Ag = 20	23 °C, 300 rpm, S/L ratio 10 g/L, 24 h	H_2_O_2_	Carboxylic acid-based DESs, particularly when combined with H_2_O_2_, efficiently leach Cu (> 80%) and Ag from waste PCBs, with selectivity influenced by DES composition. Choline chloride enhances metal complex stability, supporting DES optimization for effective e-waste metal recovery.	^21^
ChCl: FOR	Cu = 74 Ag = 18		-		
ChCl: LA	Cu = 62 Ag = 10		H_2_O_2_		
ChCl: LA	Cu = 84 Ag = 12		-		
ChCl: OX	Cu = 1 Ag = 42		H_2_O_2_		
ChCl: OX	Cu = 3 Ag = 42		-		
ChCl: ML	Cu = 75 Ag = 18		H_2_O_2_		
ChCl: ML	Cu = 68 Ag = 18		-		
ChCl: MOA	Cu = 89.5 Ag ≈ 100 Sn = 77.2	100 °C, 200 rpm, S/L 40 g/L, 6 h	Without oxidant	We demonstrate an environmentally friendly method for copper recovery from PCBs using a choline chloride–malonic acid DES, with process optimization by Taguchi design, achieving high efficiency without added oxidant agents.	This study

EG: ethylene glycol, Acid acetic: AA, MOA: Malonic acid, CA: citric acid, LA: Lactic acid, FOR: Formic acid, ML: Malic acid, N.R. not reported.

### Characterization

#### XRD and SEM

**Fig. 6:**
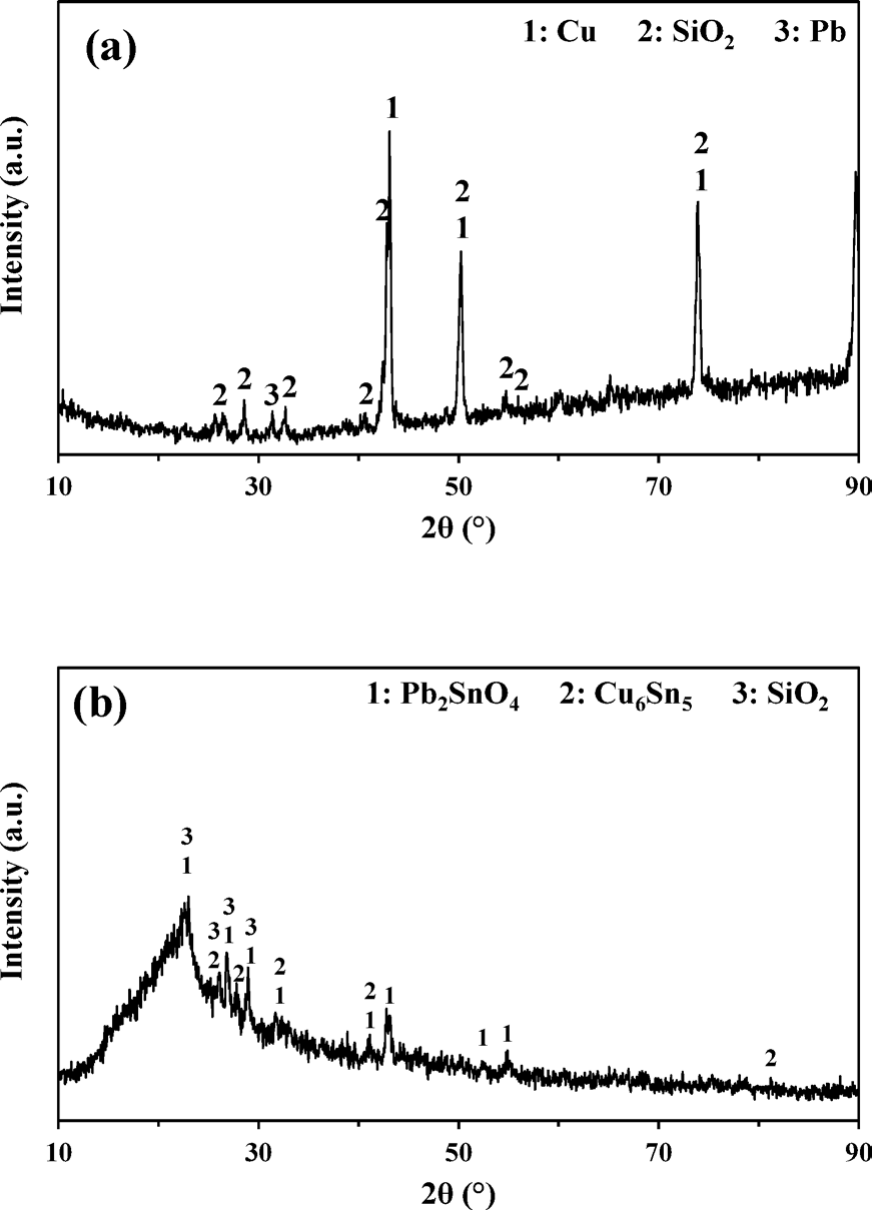
XRD pattern of **(a)** PCB and **(b)** leaching residue obtained under optimal conditions: temperature of 100 °C, leaching time of 360 min, PCB/DES ratio of 0.04 g/g, and agitation speed of 200 rpm.

The XRD profile displayed in Fig. 6 presents the phase characterization of a PCB and leaching residue under optimal conditions. According to Fig. 6a, the profile of the PCB reveals the crystalline composition of the PCB material, with distinct peaks labeled according to three main phases: copper (labeled as “1”), SiO_2_ (labeled as “2”), and Pb (labeled as “3”). The pattern shows prominent Cu peaks at approximately 43° and 50°, while SiO_2_ peaks appear at multiple positions, including around 27°, 36°, 55°, and 66°. Lead is detected at approximately 30°, indicating the complex nature of the PCB composition. The peaks were identified using reference patterns from crystallographic databases (Cu: 00–004-0836, SiO_2_: 01–076-1805, Pb: 01–087-0663), confirming the presence of these materials, which are typical components in electronic circuit boards - copper serving as the main conductive element, silicon dioxide as part of the substrate material, and lead from solder connections or other electronic components. According to Fig. 6b, the XRD profile of PCB leaching residue obtained using DES under optimal conditions (100 °C temperature, 360 min leaching time, 0.04 g/g PCB/DES ratio, and 200 rpm agitation speed), shows a broad hump or elevated background intensity is observed between 10° and 35° 2θ, which is characteristic of an amorphous phase^48,49^. The diffractogram displays the crystalline phases remaining after the leaching process. Three main phases are identified in the residue: Pb_2_SnO_4_, Cu_6_Sn_5_, and SiO_2_. The most prominent peaks appear at approximately 23° (overlapping SiO_2_ and Pb_2_SnO_4_), 30° (Cu_6_Sn_5_ and Pb_2_SnO_4_), and 47° (Pb_2_SnO_4_), with the relative peak intensities indicating the comparative abundance of these phases in the residue. The presence of these compounds suggests that while copper may have been significantly extracted under the optimal leaching conditions, tin-containing compounds and silica from the PCB substrate remain largely undissolved, demonstrating the selective nature of the DES leaching process towards different components in the PCB.

Figure S1 shows the SEM micrograph of the shredded PCB sample, highlighting the morphology, particle size, and distribution after mechanical processing. The image reveals irregularly shaped particles with rough surfaces, indicating the heterogeneous nature of PCB residues. The particle size is highly variable, ranging from a few micrometers up to several tens of micrometers, with a predominance of fine particles below ~ 20 μm. The distribution size is relatively broad, suggesting that the comminution process produces both coarse fragments and ultrafine particles. This wide size distribution is characteristic of PCBs due to the mixture of brittle ceramic/glass fibers, ductile metallic phases, and polymeric components, which fracture differently during milling. Figure S2 shows the SEM image and corresponding EDS elemental mapping of the shredded PCB sample. The EDS elemental maps (C, O, Si, Ca, Fe, Ni, Cu, Ag, Sn, Pb) reveal the distribution of the major and minor elements in the PCB. High intensities of C and O confirm the polymeric resin matrix, while Si and Ca are mainly associated with glass fibers. Cu appears in concentrated regions, corresponding to metallic conductor domains, whereas Ag, Sn, and Pb are scattered in fine inclusions, suggesting solder residues. The relative abundance obtained from the quantitative EDS analysis (Table S1) shows that C (35.99 wt%), O (22.32 wt%), Si (15.41 wt%), and Al (13.75 wt%) dominate the sample, reflecting the non-metallic matrix. Metallic constituents such as Cu (2.73 wt%), Ag (0.48 wt%), Sn (0.78 wt%), and Pb (0.92 wt%) are present in lower proportions but are economically valuable for recovery.

**Fig. 7:**
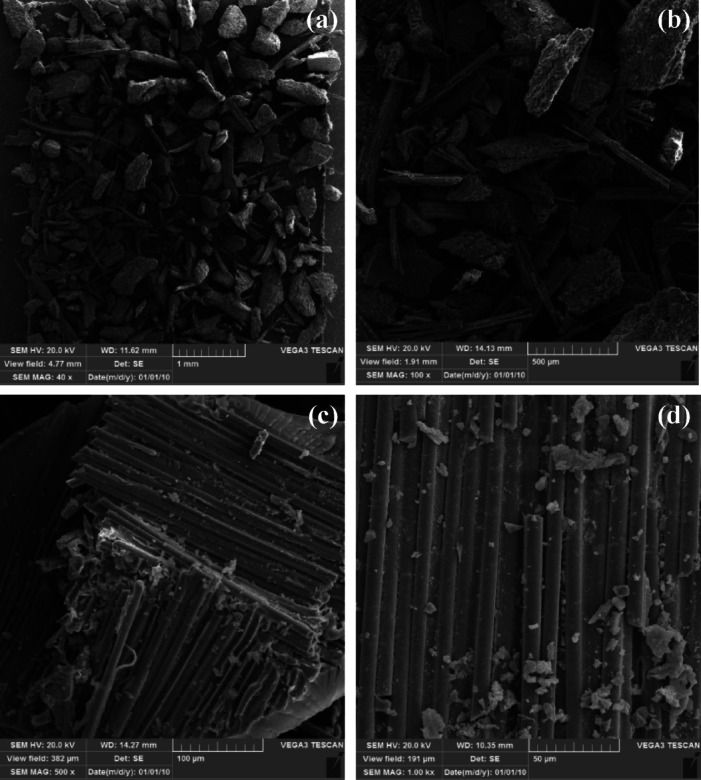
SEM image of leaching residue under optimal conditions at different magnification (temperature of 100 °C, leaching time of 360 min, PCB/DES ratio of 0.04 g/g, and agitation speed of 200 rpm).

Figure 7 shows the SEM micrographs of the leaching residue of PCB after treatment in the DES under optimal conditions (100 °C, 360 min, PCB/DES ratio of 0.04 g/g, and 200 rpm agitation speed) at different magnifications: (a) ×40, (b) ×100, (c) ×500, and (d) ×1000. At low magnifications (×40 and ×100), the residues exhibit irregular and heterogeneous morphologies, with particle sizes ranging from several hundred micrometers down to tens of micrometers. The broad particle size distribution indicates incomplete uniform fragmentation, which is typical of PCB residues composed of mixed polymeric, metallic, and ceramic phases. Larger angular fragments are accompanied by elongated fibrous structures, corresponding to glass fibers that remain intact after leaching. At higher magnifications (×500 and ×1000), the surface morphology is more clearly resolved, showing exposed glass fibers aligned in bundles. The fibers appear relatively clean, with only small residual particles adhered to their surfaces, suggesting that most of the metallic coatings and soldering alloys were effectively dissolved during the leaching process. The presence of fine adhered particles (< 5 μm) on the fiber surfaces indicates incomplete detachment of certain insoluble phases (like Cu_6_Sn_5_).

**Fig. 8:**
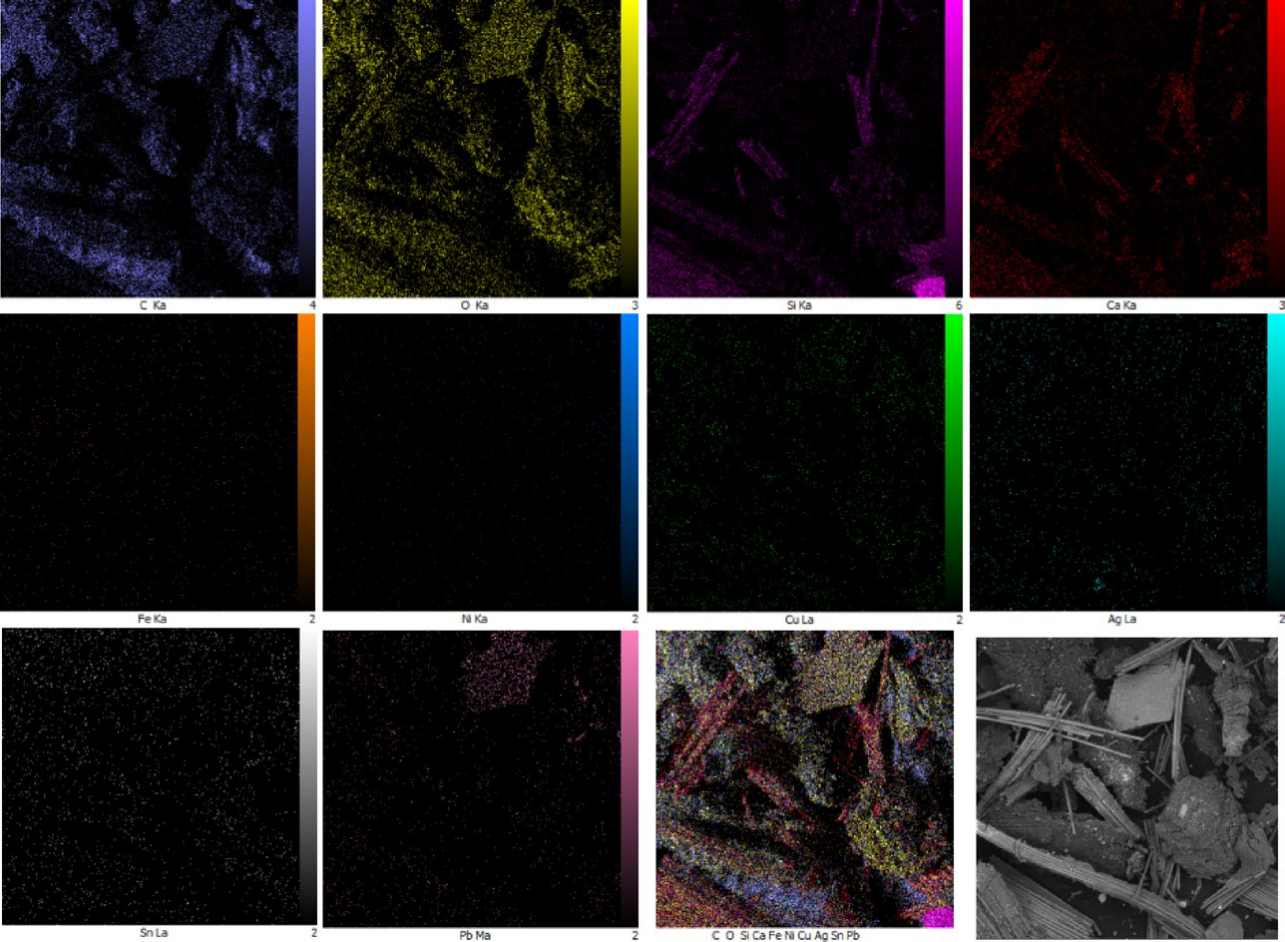
SEM-EDS analsysis of leaching residue under optimal conditions (temperature of 100 °C, leaching time of 360 min, PCB/DES ratio of 0.04 g/g, and agitation speed of 200 rpm).

Figure 8 shows the SEM-EDS elemental mapping of the leaching residue of PCB after treatment in the DES under optimal conditions. The distribution maps indicate that C and O are dominant and uniformly dispersed over the surface, reflecting the presence of organic matrix and oxidized phases. Al and Si are also clearly visible and appear in clustered regions, corresponding to glass fiber reinforcement that remains largely unaffected by leaching. Ca, Fe, Ag, and Ni appear only in trace amounts with scattered signals, suggesting partial removal during leaching. In contrast, Cu, Sn, and Pb are still detected in measurable amounts and show localized enrichments. The combined elemental map confirms that although a large fraction of metallic species was dissolved, Cu, Sn, Ag, and Pb phases persist in specific regions of the residue. This is consistent with the quantitative EDS analysis as shown in Table 7, where Sn (3.17 wt%), Cu (1.26 wt%), Ag (1.13 wt%), and Pb (1.10 wt%) remain. When compared to the untreated PCB composition (Table S1), a substantial reduction of Ca, Fe, and Si is observed, while the retention of Sn, Pb, and Cu corroborates the XRD results indicating that Pb_2_SnO_4_ and Cu_6_Sn_5_ phases are not completely leached in the DES.

**Table 7:** Eelemental anlaysis of leaching residue under optimal conditions (temperature of 100 °C, leaching time of 360 min, PCB/DES ratio of 0.04 g/g, and agitation speed of 200 rpm).

Element	C	O	Al	Si	Ca	Fe	Ni	Cu	Ag	Sn	Pb
Content (wt%)	48.58	27.51	9.42	6.38	1.21	0.17	0.07	1.26	0.01	3.17	1.10

#### FTIR

**Fig. 9:**
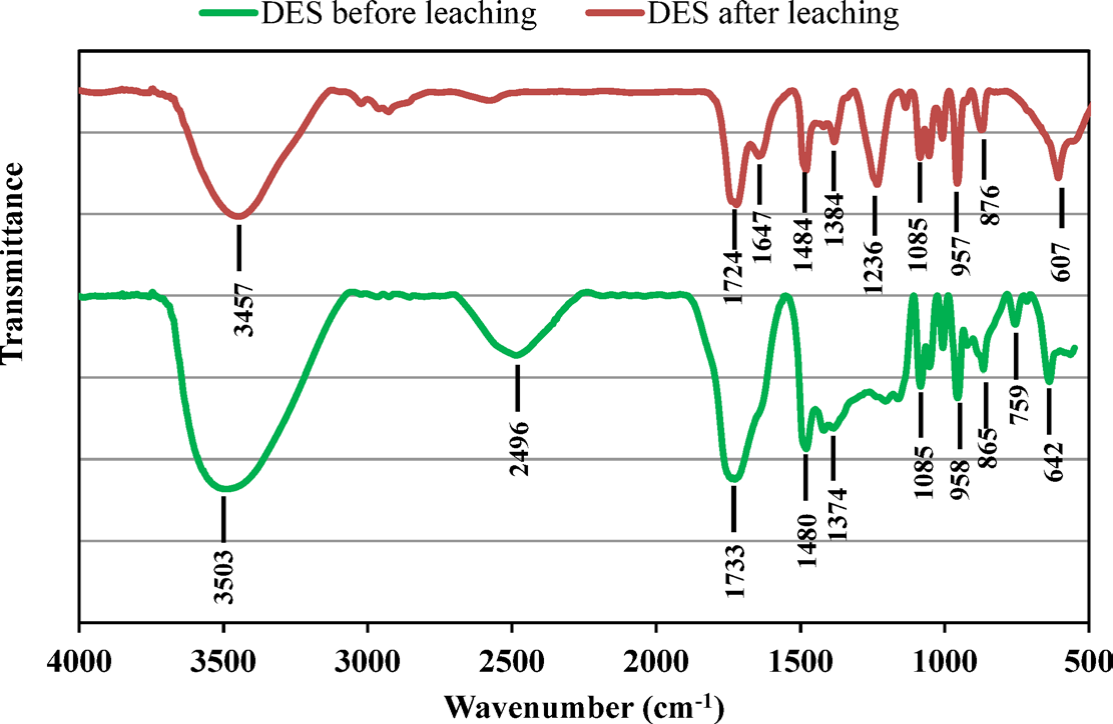
FTIR analysis of DES before and after the leaching process (temperature of 100 °C, leaching time of 360 min, PCB/DES ratio of 0.04 g/g, and agitation speed of 200 rpm).

The FTIR spectrum of the DES composed of ChCl and MOA displays several characteristic peaks that confirm hydrogen bonding interactions between the components (Fig. 9). The broadband at 3503 cm^–1^ indicates the formation of hydrogen bonds between ChCl and MOA (H − O···H, N − H···O, and O − H···Cl)^50^. The region around 2496 cm^–1^ is generally associated with the stretching vibrations of hydrogen-bonded O–H groups in carboxylic acids, particularly those involved in strong hydrogen bonds, or with overtones and combination bands^50^. The strong absorption at 1733 cm^–1^ corresponds to the C = O stretching vibration of the carbonyl compound, which has shifted from its original position (~ 1709 cm^–1^) in pure MOA due to DES formation^50,51^. The peak at 1480 cm^–1^ is attributed to CH_2_ bending vibrations of alkyl groups in ChCl^34,52,53^. The bands at 1208 and 1169 cm^–1^ are assigned to C − O stretching vibrations, with the peak near 1162 cm^–1^ specifically attributed to C − O stretching of aliphatic ketone groups. The peaks at 1085 and 1046 cm^–1^ correspond to C − N stretching of the quaternary ammonium group of choline chloride. The band at 958 cm^–1^ represents the asymmetric stretching vibration of the − CCO bond, while the peak at 865 cm^–1^ is assigned to C − N⁺ symmetric stretching^31^. The peaks at 759 cm^–1^ and 642 cm^–1^ likely correspond to various bending and deformation modes in the DES structure.

The FTIR spectrum of DES (ChCl + MOA) after leaching of PCB shows several characteristic peaks reflecting both the DES structure and possible metal interactions. The broad absorption band at 3457 cm^–1^ indicates O − H stretching vibrations associated with hydrogen bonding, which has shifted from its typical position in fresh DES due to interactions with leached metals^54^. The peaks at 3023 cm^–1^ correspond to C − H stretching vibrations from methyl groups in choline chloride.

The disappearance of the 2496 cm^–1^ band in the FTIR spectrum of a DES upon addition of metal ions is attributed to the disruption of the original hydrogen-bonding network between MOA and ChCl, specifically involving the O–H groups of malonic acid. In pure DES, the broad O–H stretching band is a hallmark of strong hydrogen bonding between the carboxylic acid groups of MOA and the chloride anions from ChCl, with the 2496 cm^–1^ feature representing the lower-energy portion of this hydrogen-bonded O–H stretch^55^. When metal ions are introduced, they coordinate with the deprotonated carboxylate groups of MOA, forming metal-carboxylate complexes. This coordination process removes the O–H protons responsible for the 2496 cm^–1^ absorption, leading to the disappearance of this band. Concurrently, the carbonyl stretching region (C = O) also shifts to lower wavenumbers due to the electron density redistribution upon metal binding, further confirming the formation of metal–carboxylate complexes and the breakdown of the original hydrogen-bonded structure^24^. The strong absorption at 1724 cm^–1^ is attributed to C = O stretching of the carbonyl groups in MOA, slightly shifted from their original position due to interactions with metal ions during leaching. The peak at 1647 cm^–1^ likely represents altered carbonyl stretching resulting from metal complexation. The band at 1484 cm^–1^ corresponds to CH_2_ bending vibrations of alkyl groups in ChCl, while 1384 cm^–1^ shows additional methyl deformations. The peaks at 1236 cm^–1^, 1134 cm^–1^, and 1057 cm^–1^ are attributed to C − O stretching vibrations and C − N stretching of the quaternary ammonium group. The band at 1008 cm^–1^ represents various C − O and C − C stretching modes. The peak at 957 cm^–1^ corresponds to the asymmetric stretching vibration of the − CCO bond, 876 cm^–1^ is assigned to C − N⁺ symmetric stretching, and the band at 607 cm^–1^ likely indicates metal-ligand interactions or other deformation modes resulting from the leaching process^56,57^.

#### UV-Vis results

**Fig. 10:**
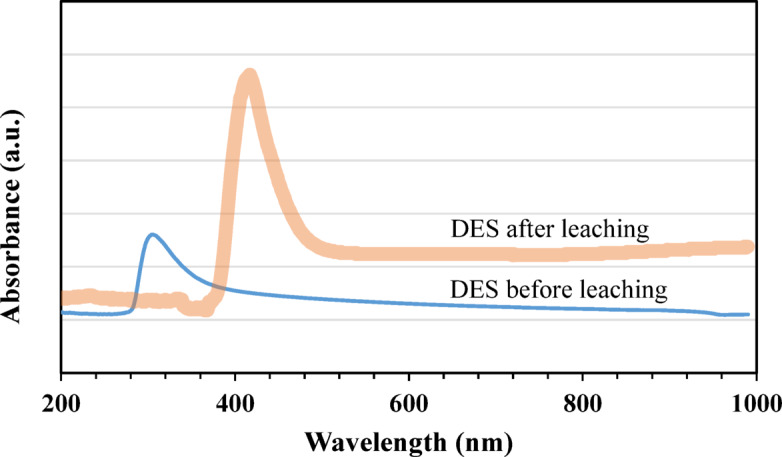
UV-Vis Spectra of ChCl + MOA DES before and after PCB leaching under optimal conditions (temperature of 100 °C, leaching time of 360 min, PCB/DES ratio of 0.04 g/g, and agitation speed of 200 rpm).

Figure 10 shows the UV-Vis Spectra of ChCl: MOA DES before and after PCB leaching under the optimal conditions. Before leaching, the spectrum of the pure DES shows a minor peak at 313 nm, which is characteristic of the background absorbance of the DES matrix. The UV-vis spectrum of the DES (ChCl + MOA) after PCB leaching shows a prominent absorption peak at 419 nm with a relatively flat profile at higher wavelengths, indicating the presence of Cu(II) species in the solution. This absorption maximum is characteristic of tetrachlorocuprate (II) complexes [CuCl_4_]^2–^ formed when Cu^2+^ ions coordinate with chloride ions available from choline chloride in the DES^58^. The absence of significant absorption bands in the 600–800 nm region, which would be typical for octahedral Cu^2+^ aquo complexes, further supports the formation of chloride-coordinated copper species rather than hydrated ones. This spectral profile aligns with previous studies of copper dissolution in choline chloride-based DES systems, where EXAFS and UV-vis spectroscopy have shown that Cu^2+^ preferentially forms chloro complexes in environments with high chloride concentration^53,58^. The formation of these chlorocopper (II) complexes contributes to the efficient leaching of copper from PCBs, allowing the ChCl: MOA DES to achieve copper recovery rates approaching 89.5%, with the distinctive color change of the solution (as shown in Fig. 10) serving as a visual indicator of successful copper extraction. It should be noted that the Cu(II)-dicarboxylate complexes show weak d-d transitions in the visible region, with absorption bands around 700 nm^59^. This spectroscopic evidence supports the hypothesis that copper leaching into the DES occurs via coordination with chloride ions from choline chloride, forming stable CuCl_4_^2–^ species. These findings correlate strongly with the results discussed in the MD simulation section, where chloride ions were shown to play a critical role in coordinating with Cu^2+^ ions, leading to stable complexation in solution.

### Molecular dynamics (MD) results

#### System equilibration and validation of simulation protocol

To elucidate the molecular-level behavior governing the recovery of Cu^2+^ ions in a DES composed of ChCl and MOA, MD simulations were performed. Given the inherent complexity and practical limitations associated with explicitly simulating the leaching process, the simulations focused on the structural and dynamic properties of the solvent matrix itself. After equilibration, RDF analyses were conducted to quantify spatial correlations and interaction patterns between atomic species within the DES.

**Fig. 11:**
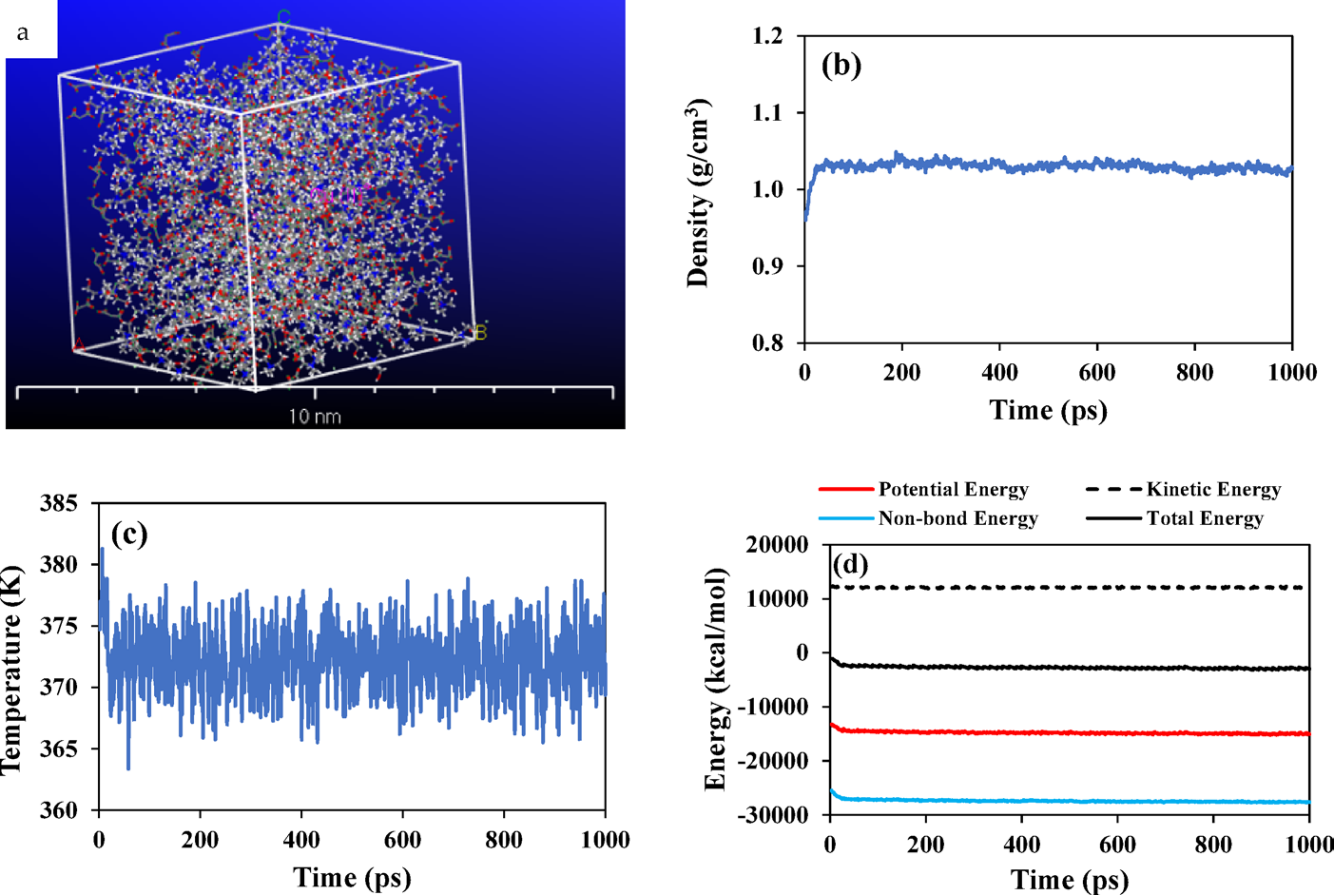
**(a)** Simulated amorphous cell, and changes in **(b)** density, **(c)** temperature, and **(d)** energy over time.

Figure 11a presents the simulated amorphous cell representing the ChCl: MOA DES. The system exhibits thermodynamic and mechanical stability throughout the simulation trajectory. The density curve stabilizes rapidly, indicating successful barostat coupling and mechanical equilibration (Fig. 11b). Based on Fig. 11c, the temperature fluctuates within a narrow range (370–380 K), reflecting proper thermal equilibration under the applied thermostat. Figure 11d presents the potential, kinetic, and total energy profiles, confirming that the system maintains energetic stability throughout the simulation. The absence of significant deviations or non-physical fluctuations indicates proper energy conservation and effective ensemble control under NPT conditions. These results confirm that the system is well-equilibrated and suitable for subsequent structural and dynamical analyses.

#### RDF analysis of hydrogen bond in DES

**Fig. 12:**
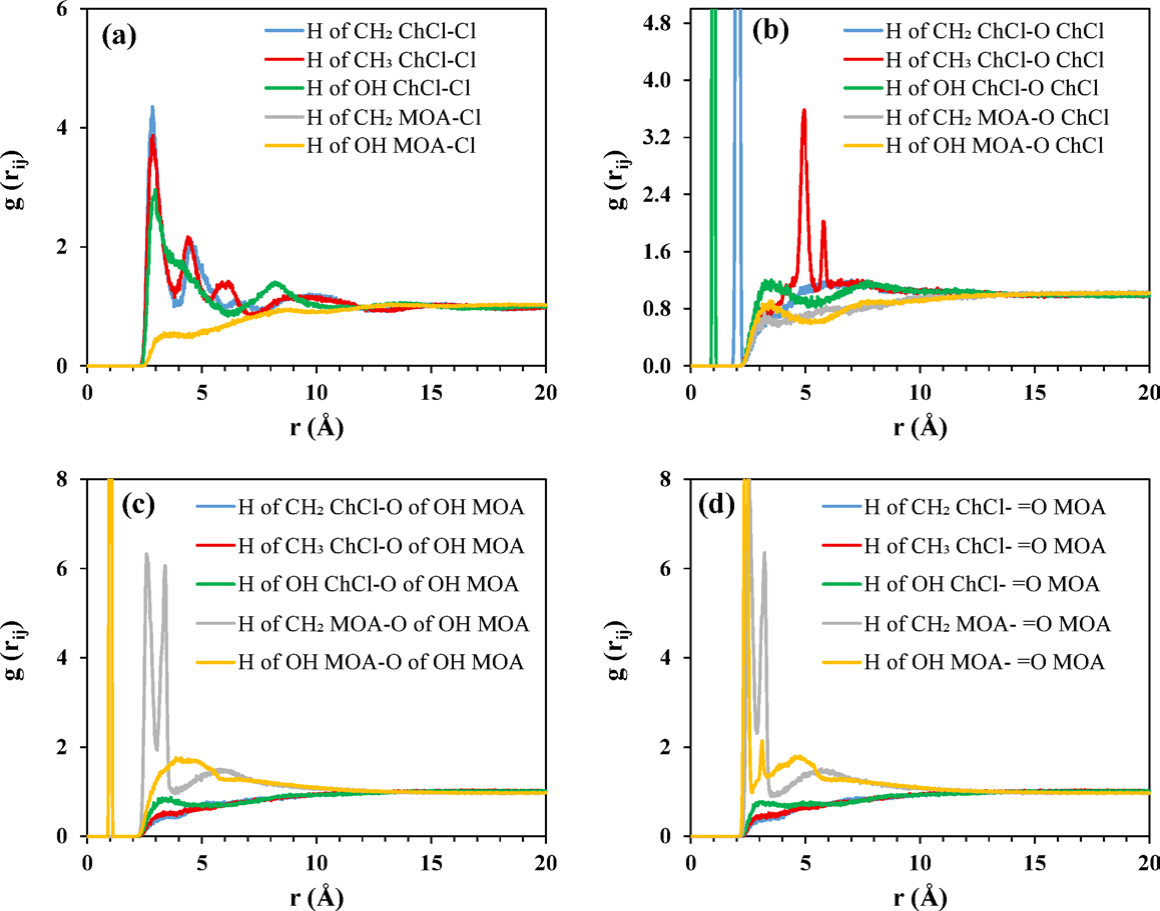
RDF curve between the hydrogen of ChCl with different groups of **(a)** ChCl and **(b)** MOA.

Figures 12a–d illustrate the RDF profiles for hydrogen atoms from distinct functional groups in ChCl and MOA concerning electronegative centers, including Cl^–^, the O atom of ChCl, and both hydroxyl and carbonyl oxygens of MOA. In Fig. 12a, the hydrogen atoms of the CH_2_ and CH_3_ groups in ChCl exhibit prominent interactions with Cl^–^, with peaks at *r* ≈ 2.87 Å (g(r) ≈ 4.25) and *r* ≈ 2.81 Å (g(r) ≈ 3.8), respectively. The OH hydrogen of ChCl also shows a notable association with Cl^–^ at *r* ≈ 2.93 Å, g(r) ≈ 2.9, suggesting participation in the hydrogen-bond network.

The hydrogen of the OH group in MOA interacts with Cl^–^ at *r* ≈ 3.73 Å (g(r) ≈ 0.5), which, while less intense, still indicates a meaningful hydrogen-bonding contribution. This suppression of MOA the hydrogen–chloride interaction may be attributed to two key structural effects. First, the presence of Cu^2+^ ions, introduced during PCB leaching, results in strong electrostatic coordination with both Cl^–^ and carboxylate oxygens of MOA, thereby reducing the availability of free chloride ions for hydrogen bonding. Second, MOA molecules tend to engage in intramolecular hydrogen bonding or form cyclic dimers via their two carboxylic acid groups, effectively shielding the OH hydrogens from external acceptors. These internal interactions are reflected in Figs. 12c and d, where intense RDF peaks are observed for CH_2_ of MOA interacting with OH of MOA at *r* ≈ 2.61 and 3.41 Å, and for OH of MOA with = O of MOA at *r* ≈ 2.41, indicating strong localized hydrogen bonding.

In Fig. 12b, which represents RDFs between hydrogen atoms and the oxygen atom of the OH group in ChCl, the most prominent peak is observed for the CH_3_ of ChCl at *r* ≈ 4.97 Å with g(r) ≈ 3.5, indicating a weak, long-range spatial correlation rather than a directional hydrogen bond. Other hydrogen atoms, including CH_2_ and OH from both ChCl and MOA, exhibit only minor peaks in the range of *r* ≈ 3.8–5.5 Å with g(r) < 1.5, suggesting that the hydroxyl oxygen of choline is not a dominant hydrogen-bond acceptor in this system. This may be attributed to steric hindrance around the hydroxyl group of choline or competition with stronger acceptor groups, such as the chloride anion or the carboxylate oxygen of MOA^60^. Moreover, the influence of metal ions such as Cu^2+^ may further suppress these interactions by restructuring local solvation shells.

In contrast, Fig. 12c, which presents RDFs for hydrogen atoms around the hydroxyl oxygen of MOA, displays a distinctly different pattern. The OH hydrogen of MOA shows a sharp and intense peak at *r* ≈ 1.01 Å, indicative of a strong intramolecular hydrogen bond. This very short distance corresponds to covalently bound hydrogen or a tightly constrained hydrogen bond within the MOA molecule. The two distinct RDF peaks observed between the CH_2_ hydrogen atoms and the oxygen of the OH groups in the MOA (at *r* ≈ 2.61 Å and *r* ≈ 3.37 Å) are likely a consequence of the presence of two carboxylic acid groups in the MOA molecule, positioned symmetrically on either side of the central methylene (CH_2_) group. This molecular arrangement allows the CH_2_ hydrogens to engage in non-equivalent hydrogen bonding interactions with both OH groups, depending on conformational orientation and local solvation structure. These dual features support the formation of intermolecular MOA–MOA associations, such as cyclic dimers or extended hydrogen-bonded clusters, and reflect the complex solvation environment within the DES. Consequently, these internal interactions can reduce the availability of MOA hydrogen bond donors for interactions with other components, including Cl^–^ or ChCl^61,62^.

Figure 12d, which presents RDFs between various hydrogen atoms and the carbonyl oxygen (= O) of MOA, further confirms the prevalence of MOA–MOA self-association. The OH hydrogen of MOA dominates the RDF profile with two distinct peaks, suggesting strong and directional hydrogen bonding, likely involving either intermolecular association with neighboring MOA molecules or intramolecular stabilization. Notably, the CH_2_ hydrogens of MOA also exhibit two pronounced RDF peaks, as shown more clearly in Fig. 10d. These peaks reflect non-equivalent hydrogen bonding interactions with the = O group, which can be attributed to the presence of two adjacent carboxylic acid groups in MOA. The molecular geometry allows CH_2_ to form hydrogen bonds with either carbonyl group under different conformations, giving rise to the two-peak structure. These interactions strongly support the existence of cyclic dimers or hydrogen-bonded clusters of MOA in the DES matrix^63^. In contrast, hydrogen atoms from ChCl, including those from CH_2_, CH_3_, and OH groups, exhibit broad and low-intensity g(r) profiles in this region, indicating a minimal interaction with the carbonyl oxygen of MOA.

The RDF results obtained in this study strongly agree with previous findings on DES structure and hydrogen bonding behavior. Gautam et al^62^. reported that in ChCl–carboxylic acid systems, both the hydroxyl hydrogen of choline and the acidic hydrogen of the organic component contributed comparably to chloride coordination, reflecting a balanced hydrogen-bonding network. In contrast, our results indicate that hydrogen atoms from choline maintain strong interactions with chloride. At the same time, the contribution from MOA is less pronounced, likely due to internal hydrogen bonding and competitive coordination with copper ions^64^. Previous studies demonstrated that transition metal ions, particularly Cu^2+^, disrupt hydrogen bond networks in DESs through strong metal–ligand complexation and chloride sequestration^65^.

#### RDF analysis of copper bond in DES

**Fig. 13:**
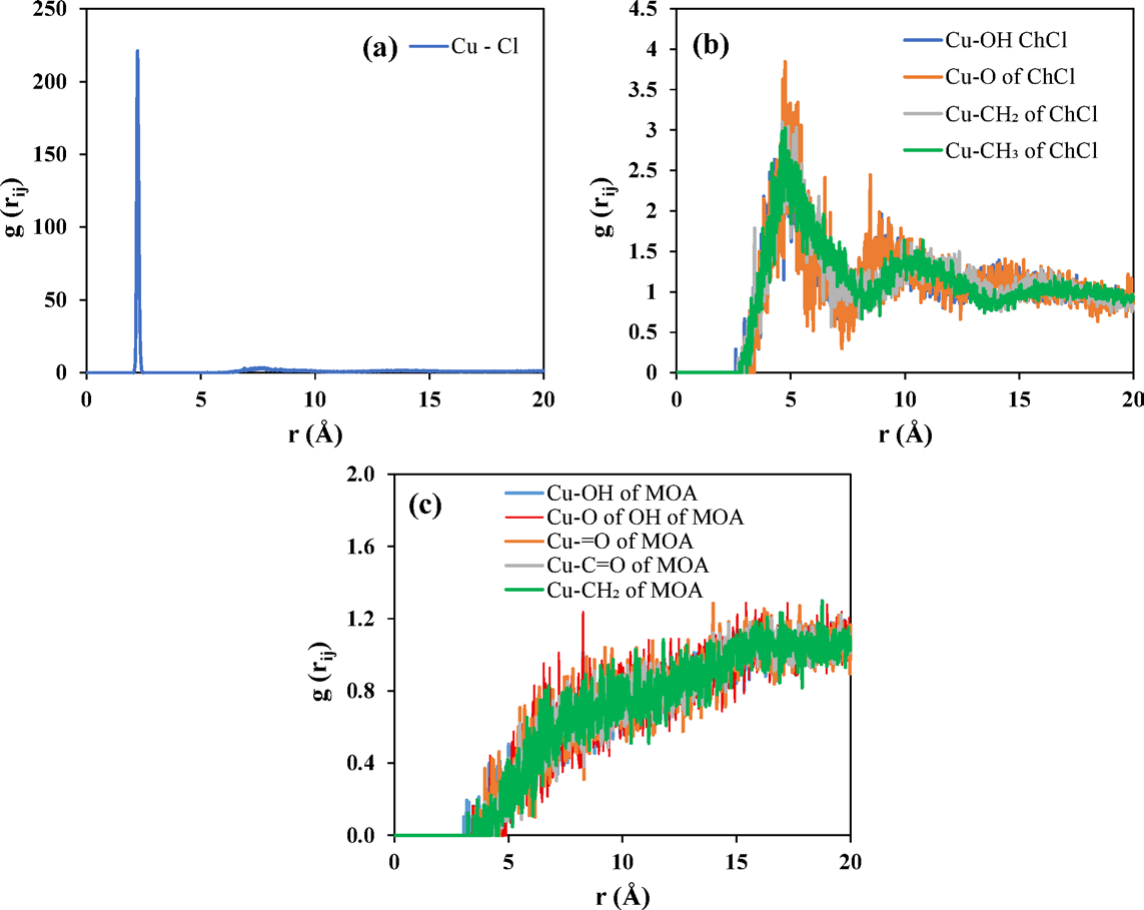
RDF results between copper ions with **(a)** Cl of ChCl, **(b)** different groups of ChCl, and **(c)** different groups of MOA.

In Fig. 13, the RDF curves of Cu ions with different components of ChCl and MOA in DES solutions reveal valuable insights into the atomic-level interactions between these components. The RDF curve (Fig. 13a) for Cu interactions with Cl in ChCl shows a distinct peak, indicating a strong coordination between Cu ions and Cl anions in the DES. The sharpness of the peak in the RDF curve implies a well-structured and ordered coordination environment around the Cu ion, indicating that chloride ions are closely associated with the Cu ion at a specific distance (at around 2.23 Å). This behavior is typical for Cu ions, which tend to form ionic bonds with halide ions like chloride^29,31,33,66^. Similarly, in a binary DES composed of choline chloride and p-toluenesulfonic acid (PTSA), molecular dynamics simulations confirmed the formation of stable [CuCl_4_]^2–^-like complexes via strong Cu^2+^–Cl^–^ coordination, which facilitated chalcopyrite dissolution and enhanced metal extraction efficiency^33^.

Based on Fig. 13b, the RDF data for Cu-OH, Cu-O, and Cu-CH_2_ interactions in ChCl show a clear preference for Cu ions to coordinate with the oxygen atoms of the hydroxyl (OH) groups and the oxygen atoms in the ChCl structure. The presence of distinct peaks in the RDF for Cu-O and Cu-OH interactions indicates that these coordination bonds are strongly favored over interactions with the nonpolar alkyl groups. This behavior is typical for Cu ions, which have a high affinity for oxygen donors, forming stable coordination complexes in polar solvents like ChCl. The relatively weaker peaks observed in the RDF for Cu-CH_2_ and Cu-CH_3_ interactions suggest that these alkyl groups are less involved in coordinating with Cu ions, as expected from the lower electronegativity of carbon atoms compared to oxygen^30^.

Figure 13c illustrates the RDFs between Cu^2+^ ions and various atomic sites in MOA, including OH, O of OH, =O, C = O, and CH_2_. The RDF curves for all five interactions show no distinct peaks within the examined distance range, and the g(r) values remain low and gradually increasing, indicative of non-specific, diffuse interactions rather than well-defined coordination. The absence of any sharp or structured maxima confirms that Cu^2+^ does not form stable complexes with any functional group of MOA. These results clarify that, contrary to previous assumptions, MOA does not significantly contribute to Cu^2+^ complexation in the DES phase during PCB leaching. Unlike in systems where carboxylic acid groups participate in bidentate coordination, the RDF trends show that neither OH nor = O atoms in MOA act as coordinating ligands for Cu^2+^. Instead, Cu^2+^ coordination is more likely dominated by Cl^–^, consistent with forming [CuCl_4_]^2–^-like complexes as observed in other ChCl-based DES systems. The weak and structureless RDF profiles for CH_2_ also support this conclusion, as alkyl hydrogens are not expected to contribute to metal coordination.

The coordination number (CN) analysis further confirms the dominant role of chloride ions in copper solvation within the ChCl: MOA DES system. The CN of Cl^–^ around Cu^2+^ is estimated to be approximately 4.45 at a distance of 2.67 Å, indicating a well-defined tetrahedral [CuCl_4_]^2–^ coordination environment. This strongly supports the formation of discrete anionic complexes, consistent with previous MD and spectroscopic studies of chloride-rich systems. In contrast, the CN between Cu^2+^ and the oxygen atoms of ChCl is significantly lower, around 2.1, and the CN for the OH group is approximately 2.01, both occurring at longer distances near 7.5 Å, which suggests weak, second-shell interactions rather than direct coordination. These results highlight the preferential binding of Cu^2+^ to Cl^–^ over oxygen-containing groups, reinforcing the conclusion drawn from RDF profiles that MOA does not participate in Cu^2+^ complexation, and that the primary coordination environment for Cu^2+^ in this DES is dictated by halide ligation.

Overall, the combined MD and UV–Vis results conclusively demonstrate that copper speciation in the ChCl: MOA DES is dominated by chloride coordination, with Cu^2+^ forming stable [CuCl_4_]^2–^ complexes. The UV–Vis spectrum after PCB leaching exhibits a distinct absorption band at 419 nm, characteristic of chlorocuprate(II) species, while the absence of peaks in the 600–800 nm region excludes the formation of hydrated or dicarboxylate-bound Cu^2+^ complexes. These spectroscopic observations strongly support the MD-derived coordination number of ~ 4 for Cu–Cl interactions, highlighting the central role of chloride ligands in governing copper solvation and reactivity in halide-based DESs.

### Leaching mechanism and copper separation

The dissolution mechanism occurs through an oxidation-complexation process, where Cu^2+^ ions are extracted from the PCB material and form stable copper-chloride complexes in the DES, as indicated by both UV-vis analysis and MD simulations. These analyses revealed the presence of Cu(II) species in the form of tetrachlorocuprate (II) complexes [CuCl_4_]^2−^, formed by Cu^2+^ ions coordinating with chloride ions from ChCl in the DES. Previous research has also observed the formation of divalent copper complexes, further supporting the idea that copper tends to form chloride-coordinated complexes in environments with high chloride concentrations^67^. Based on these findings, a proposed reaction mechanism is:

2 $$\begin{array}{*{20}{l}} {\mathrm{Cu}\left(\mathrm{s} \right){\text{ }} + {\text{ }}4\mathrm{Cl}{^-} \to {\text{ }}{{\left[ {\mathrm{CuCl}{_4}} \right]}^{2-}}\left( {\mathrm{in}{\text{ }}\mathrm{DES}} \right).} \end{array}$$

Following the leaching stage, the recovery of copper from DES)solutions is a critical step that leverages established hydrometallurgical techniques to ensure both efficiency and sustainability. The primary methods for copper recovery from DES include electrochemical techniques, precipitation, and solvent extraction, each offering distinct advantages depending on the composition of the leach solution and the desired end product. Electrochemical methods, encompassing both cementation and electrodeposition, are widely employed for copper recovery from DESs due to their high efficiency and selectivity. Mishra et al^20,54^. demonstrated the effectiveness of cementation by using zinc powder to recover copper from a choline chloride-formic acid (FA ChCl) DES, achieving an impressive recovery efficiency of 99.8% from thermally treated PCBs. Although the study did not specifically investigate DES regeneration, it confirmed that the solvent could maintain high performance over multiple leaching cycles, underscoring its reusability. In a different approach, Liu et al^20^. coupled leaching with electrodeposition in a ChCl-EG DES, using hydrogen peroxide as an oxidizing agent to extract copper from waste PCBs. This method achieved up to 97.8% copper recovery, and the regenerated DES exhibited excellent potential for reuse in subsequent cycles, highlighting its sustainability and low degradation. Further supporting this technique, Zhang and Hau^68^explored the electrochemical deposition of copper from Cu(I) oxide in a ChCl-urea DES, emphasizing the viability of electrowinning for direct metal recovery. Additionally, Mandroyan et al^69^. investigated the electrochemical reduction of copper salts in a ChCl-ethylene glycol DES, finding that factors like temperature and ultrasound could enhance mass transfer and reduction efficiency, which indirectly supports the potential for DES reuse across multiple cycles.

Precipitation is another effective strategy for selectively recovering copper from DES solutions, often yielding high-purity products that can be easily separated. Zhao et al^4^. employed a two-stage process using a ChCl-GA DES for copper extraction from WPCBs. After leaching, copper was precipitated from the loaded DES by adding an oxalic acid solution, resulting in the recovery of 74.93% of the copper as copper oxalate (CuC_2_O_4_·2H_2_O) with a purity exceeding 98%. Solvent extraction serves as a powerful technique for transferring copper from the DES phase to an aqueous system, facilitating selective recovery and minimizing waste. Routray et al^70^. investigated this approach by first leaching copper from a spent catalyst and a copper concentrate using a ChCl-EG DES, achieving over 80% recovery. The loaded copper was then successfully transferred to an aqueous phase via solvent extraction with commercial extractants such as LIX-84 and Na-DEHPHA. Thus, it can be confidently concluded that methods for recovering metals from DES are available, and by isolating the metal, it can be reused. The primary objective of this study was to assess the feasibility of the dissolution process and to extract data on the underlying mechanisms.

A further point that warrants attention is the influence of water on the characteristics of DESs and how this affects process design. Research has shown that DESs can incorporate considerable amounts of water without breaking down the hydrogen-bond network, all while lowering viscosity and cutting overall process expenses. These hydrated DES systems have proven effective in extracting metals from different resources^71,72^. Although solvents like Ethaline can preserve their structure with water content up to 30 wt%, greater hydration typically leads to decreases in melting point, density, and viscosity, alongside improvements in ionic mobility, polarity, and solubility. For example, the addition of merely 10 wt% water can cut viscosity by 80% and boost conductivity threefold^27,73^. Nevertheless, this comes at the cost of disrupting hydrogen bonding, which may undermine solvent stability and, by raising toxicity, pose environmental concerns^74^. Conductivity tends to rise with hydration until it reaches an optimum near 60 wt% water, driven by enhanced ionic dissociation, after which it falls off as the system becomes overly diluted^27^.

While this study presents promising results, scaling up the DES-based copper recovery process still faces several challenges that need to be addressed in future studies. Issues such as solvent regeneration efficiency, long-term stability, and the handling of large volumes of leachate require further investigation. Nonetheless, the high efficiency and potential reusability of the DES solvent suggest that, with continued research, this method could provide a sustainable and cost-effective alternative to traditional pyrometallurgical and hydrometallurgical methods. Future work should focus on overcoming these challenges and assessing the feasibility of integrating this process into large-scale operations.

## Conclusion

This study demonstrates the effective recovery of copper from PCBs using a two-component DES composed of ChCl and MOA, without the use of an oxidant agent. The optimization of key process parameters, temperature, leaching time, and PCB/DES ratio, highlighted their significant impact on copper recovery, with temperature proving to be the most influential factor. Specifically, copper recovery increased from 20.1% at 40 °C to 93.5% at 110 °C due to enhanced reaction kinetics and reduced viscosity. Extended leaching time and lower PCB/DES ratios further contributed to improved copper extraction. The highest copper recovery of 89.5% was achieved under optimized conditions, including a temperature of 100 °C, a leaching time of 360 min, a PCB/DES ratio of 0.04 g/g, and a stirring speed of 200 rpm. The FTIR spectra confirmed the formation of metal–carboxylate complexes, and the stability of most hydrogen bonds after leaching suggests the potential reusability of the solvent. Additionally, MD simulations and UV-vis analysis demonstrated that copper coordination in ChCl: MOA DES is predominantly chloride-driven, forming stable [CuCl_4_]^2−^ complexes, with minimal interaction from MOA. These findings contribute to a deeper understanding of the copper leaching process and the role of DES in enhancing copper recovery. Although this study shows promising results, scaling up the DES-based copper recovery process faces challenges like solvent regeneration, long-term stability, and handling large volumes of leachate, which require further research to make it a sustainable and cost-effective alternative to traditional methods.

## Supplementary Information

Below is the link to the electronic supplementary material.

Supplementary Material 1

## Data Availability

Data will be made available on the request.
