# Supplementary material for: Copper recovery from printed circuit boards using an oxidant-free deep eutectic solvent: experimental and molecular dynamics approach
Source: Sci Rep. 2026 Apr 29;16:19984. doi: 10.1038/s41598-026-42446-7 (PMC13319738; doi:10.1038/s41598-026-42446-7)
Supplement: Supplementary file 1 — Supplementary Material 1 [file 41598_2026_42446_MOESM1_ESM.docx]

**Copper Recovery from Printed Circuit Boards Using an Oxidant-Free Green Deep Eutectic Solvent: Experimental and Molecular Dynamics Approach**

Pardis Bedrodian ^1^, Saeid Karimi ^1,*^ and Mojtaba Esmailzadeh ^2,*^

^1^ Department of Metallurgy and Materials Engineering, Hamedan University of Technology, Hamedan, Iran.

^2^ Department of Mechanical Engineering, Persian Gulf University, Bushehr, Iran.

**^*^**Corresponding authors: SK: [s.karimi@hut.ac.ir](mailto:s.karimi@hut.ac.ir); [karimi6439@gmail.com](mailto:karimi6439@gmail.com); M.E. [m.esmaeilzade@pgu.ac.ir](mailto:m.esmaeilzade@pgu.ac.ir)

Figure S1. SEM image of shredded PCB.

Figure S2. SEM-map image of shredded PCB.

Table S1. Elemental analysis of shredded PCB

| Element | C | O | Al | Si | Ca | Fe | Ni | Cu | Ag | Sn | Pb |
| --- | --- | --- | --- | --- | --- | --- | --- | --- | --- | --- | --- |
| Content (wt.%) | 35.99 | 22.32 | 13.75 | 15.41 | 6.40 | 1.17 | 0.05 | 2.73 | 0.48 | 0.78 | 0.92 |
